# A Giant Chelonioid Turtle from the Late Cretaceous of Morocco with a Suction Feeding Apparatus Unique among Tetrapods

**DOI:** 10.1371/journal.pone.0063586

**Published:** 2013-07-11

**Authors:** Nathalie Bardet, Nour-Eddine Jalil, France de Lapparent de Broin, Damien Germain, Olivier Lambert, Mbarek Amaghzaz

**Affiliations:** 1 CNRS UMR 7207, Département Histoire de la Terre, Muséum National d’Histoire Naturelle, Paris, France; 2 Cadi Ayyad University, Faculty of Sciences Semlalia, Department of Earth Sciences, Vertebrate Evolution and Palaeoenvironnements, Marrakech, Morocco; 3 Institut Royal des Sciences Naturelles de Belgique, Département de Paléontologie, Bruxelles, Belgium; 4 Office Chérifien des Phosphates, Centre Minier de Khouribga, Khouribga, Morocco; Team 'Evo-Devo of Vertebrate Dentition', France

## Abstract

**Background:**

Secondary adaptation to aquatic life occurred independently in several amniote lineages, including reptiles during the Mesozoic and mammals during the Cenozoic. These evolutionary shifts to aquatic environments imply major morphological modifications, especially of the feeding apparatus. Mesozoic (250–65 Myr) marine reptiles, such as ichthyosaurs, plesiosaurs, mosasaurid squamates, crocodiles, and turtles, exhibit a wide range of adaptations to aquatic feeding and a broad overlap of their tooth morphospaces with those of Cenozoic marine mammals. However, despite these multiple feeding behavior convergences, suction feeding, though being a common feeding strategy in aquatic vertebrates and in marine mammals in particular, has been extremely rarely reported for Mesozoic marine reptiles.

**Principal Findings:**

A relative of fossil protostegid and dermochelyoid sea turtles, *Ocepechelon bouyai* gen. et sp. nov. is a new giant chelonioid from the Late Maastrichtian (67 Myr) of Morocco exhibiting remarkable adaptations to marine life (among others, very dorsally and posteriorly located nostrils). The 70-cm-long skull of *Ocepechelon* not only makes it one of the largest marine turtles ever described, but also deviates significantly from typical turtle cranial morphology. It shares unique convergences with both syngnathid fishes (unique long tubular bony snout ending in a rounded and anteriorly directed mouth) and beaked whales (large size and elongated edentulous jaws). This striking anatomy suggests extreme adaptation for suction feeding unmatched among known turtles.

**Conclusion/Significance:**

The feeding apparatus of *Ocepechelon*, a bony pipette-like snout, is unique among tetrapods. This new taxon exemplifies the successful systematic and ecological diversification of chelonioid turtles during the Late Cretaceous. This new evidence for a unique trophic specialization in turtles, along with the abundant marine vertebrate faunas associated to *Ocepechelon* in the Late Maastrichtian phosphatic beds of Morocco, further supports the hypothesis that marine life was, at least locally, very diversified just prior to the Cretaceous/Palaeogene (K/Pg) biotic crisis.

## Introduction

### The Phosphates of Morocco: a Hotspot of Vertebrate Palaeobiodiversity at the K/Pg Turnover

The Late Cretaceous - Early Palaeogene Phosphates of Morocco are part of the ‘Mediterranean Tethyan Phosphogenic Province’, a large belt of sedimentary rocks deposited around palaeolatidude 20°S, outshore of the NW part of the African Craton [1]. These phosphatic deposits currently crop out widely in the Middle East, North and West Africa, up to the Pernambuco Province of Brazil, where they are exploited as a valuable economical resource.

Beyond their economical interest, the Phosphates of Morocco are characterized by their extreme richness (both in terms of taxonomical diversity and specimen abundance) in Maastrichtian to Ypresian marine vertebrate taxa, which include selachians, osteichthyan fishes, both continental and marine reptiles (including birds), as well as terrestrial mammals [2], [3]. Around 96% of the taxa found are of marine origin. Near-shore high productivity during periods of intense upwelling probably played an important role to explain this important paleobiodiversity and the plethora of described species [1].

Among these vertebrate remains, reptiles, and especially marine forms are, with selachians, the most diversified and abundant. They are represented by more than fifty species along the Maastrichtian-Ypresian Phosphatic series, which represents about one fourth of the total vertebrate species known in the Phosphates of Morocco. During the Maastrichtian, mosasaurid squamates were the most abundant and diversified, representing half the total of species found [3] (see Table 1).

**Table 1:** Maastrichtian marine reptile diversity in the Phosphates of Morocco.

ORDERS	FAMILIES	SPECIES
SQUAMATA	Palaeophidae	*Palaeophis* sp.
	Pachyvaranidae	*Pachyvaranus crassispondylus*
	Mosasauridae	*Mosasaurus beaugei*
		*Prognathodon currii*
		*Prognathodon giganteus*
		*Prognathodon* nov. species
		*Eremiasaurus heterodontus*
		*Globidens phosphaticus*
		*Carinodens belgicus*
		*Carinodens minalmamar*
		«*Platecarpus*» *ptychodon*
		*Halisaurus arambourgi*
**PLESIOSAURIA**	**Elasmosauridae**	*Zarafasaura oceanis*
		Elasmosauridae indet.
**CROCODYLIFORMES**	**Gavialoidea**	*Ocepesuchus eoafricanus*
	**Crocodyliformes**	Genus & species indet.
**CHELONII**	**Cheloniidae**	*Euclastes* sp.
		Genus & species indet.
	**Dermochelyoidae**	***Ocepechelon bouyai***
		Genus & species indet.
	**Bothremydidae**	Genus & species indet. A
		Genus & species indet. B

From [3] and personal observations (F.L.B. and N.B.) for unpublished data.

As far as chelonians are concerned, the Maastrichtian taxa remain poorly known (see Table 1) and their record is scarce compared to that of the very abundant and highly diversified Palaeogene ones [4], [5]. They include both bothremydid pleurodirans and chelonioid cryptodirans. Up to now, they are only known by a skull of the cheloniid *Euclastes* [6], a hyoplastron of an indeterminate ‘dermochelyid’ [7] that might prove to belong to the same taxon as described here or to a closely related one (see Text S1, p. 11–13, in Supporting Information S1), indeterminate forms of cheloniids and bothremydids [8] (pers. obs.), as well as a new Dermochelyoidae currently under study (Lapparent et al., in prep.) (see Table 1).

### The Sea Turtle Fossil Record

Sea turtles, the Chelonioidea (Cryptodira), are known in the fossil record since the Early Cretaceous [9] or the Late Jurassic [10], [11] and constitute the main group of modern marine reptiles. Their highest diversity occurred during the Late Cretaceous – Early Palaeogene interval, when they were represented by a large number of taxa showing diverse ecological adaptations to an aquatic life, including a large variety of predators, though being edentulous forms [12].

In Africa, Cretaceous cryptodiran turtles are rather scarce and, apart from the taxa from the Maastrichtian Phosphates of Morocco above mentioned, the only other record is *Angolachelys* Mateus et al., 2009 from the Turonian of Angola [13].

Here we describe a new genus and species of marine chelonian found in the Late Maastrichtian of the Oulad Abdoun Basin, central Morocco. It represents not only one of the best records of the rare cryptodiran turtles from the Cretaceous of Africa, but also a new giant form of Chelonioidea recorded just prior to the K/Pg biological crisis, and most notably, a taxon exemplifying a suction feeding apparatus unique among tetrapods.

## Materials and Methods

### The Phosphates of Morocco Sites

**Figure 1:**
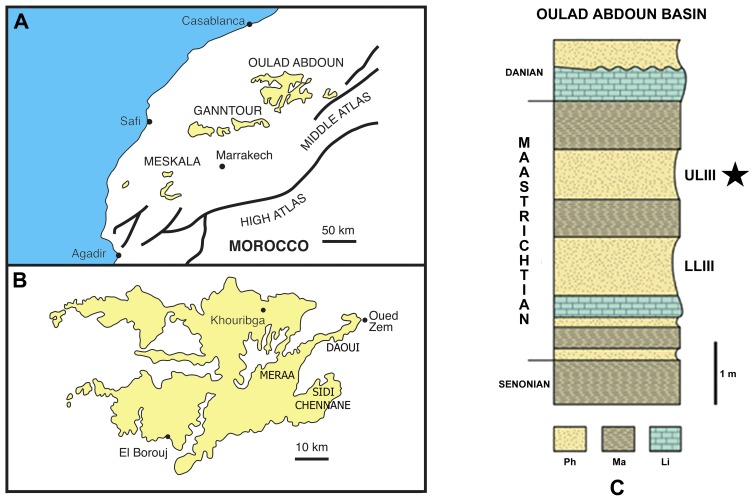
*Ocepechelon bouyai* gen. et sp. nov., geographical and stratigraphical occurrence. *Ocepechelon* has been found in the Upper Level III (Upper Maastrichtian) phosphatic deposits of the Sidi Chennane area, SE part of the Oulad Abdoun Basin, central Morocco. **A**, general geographical map of Morocco showing the main phosphatic basins (Ganntour and Oulad Abdoun basins); **B**, details of the Oulad Abdoun Basin with the northeastern main exploited areas (Daoui, Meraa El Arach, Sidi Chennane); **C**, synthetical stratigraphical column of the Maastrichtian phosphatic series in the northeastern part of the Oulad Abdoun Basin. **Abbreviations** : LLIII, Lower Level III; ULIII, Upper Level III; Ph, phosphates; Ma, marls; Li, limestones. The black star indicates the stratigraphical occurrence of *Ocepechelon bouyai* gen. et sp. nov.

The Phosphates of Morocco crop out in several basins, the most important being the Oulad Abdoun and the Ganntour basins, in central Morocco (Figure 1A). These phosphatic deposits stratigraphically extend from the Late Cretaceous (Maastrichtian) to the base of the middle Eocene (Lutetian), spanning the largest interval of all Tethyan phosphates [1]. The Maastrichtian phosphatic level of the Oulad Abdoun Basin is called *Couche III* (Level CIII). It is considerably condensed in the eastern part of the basin (Figure 1B–C), being only about 2 to 5 meters thick. It includes two exploited levels of soft phosphates: the yellowish Lower Level III (LLIII), and the greyish Upper Level III (ULIII) (Figure 1C). The series is Late (but not latest) Maastrichtian in age.

### Nomenclatural Acts

The electronic edition of this article conforms to the requirements of the amended International Code of Zoological Nomenclature, and hence the new names contained herein are available under that Code from the electronic edition of this article. This published work and the nomenclatural acts it contains have been registered in ZooBank, the online registration system for the ICZN. The ZooBank LSIDs (Life Science Identifiers) can be resolved and the associated information viewed through any standard web browser by appending the LSID to the prefix “http://zoobank.org/”. The LSID for this publication is: urn:lsid:zoobank.org:pub: DA469295-7A8F-4DD1-A59F-090FC9DB4754. The electronic edition of this work was published in a journal with an ISSN, and has been archived and is available from the following digital repositories: PubMed Central, LOCKSS.

### Phylogenetic Analysis

The phylogenetic analyses have been performed with PAUP*, NONA (winclada interface), and TNT, with all characters considered unordered. The used matrix is modified from Hiramaya [14] and Kear & Lee [15]. Several rootings have been tried: with and without the hypothetical taxon and the other outgroups. Principal results of the phylogenetical analyses are presented below, with different cladograms obtained according to the variable inclusion of the outgroups. It appears that the results are more congruent with the current knowledge of turtle evolution if the hypothetical taxon is retained in all the tested examples, and particularly for the matrix with two outgroups (hypothetical taxon and Chelomacryptodira). The inclusion of Chelomacryptodira (including both Trionychoidea and Testudinoidea) in the analysis allows the identification of potential homoplasies of a negligible importance between Chelonioidea (including *Ocepechelon*) and some Chelomacryptodira (see Text S2 in Supporting Information S1).

The main and indispensable synapomorphies of each monophyletic chelonioid group, which are clearly evidenced even if the groups are variably assembled, are presented in the Text S2 and Figure S1 in Supporting Information S1.

## Results

### Systematic Palaeontology

**Systematic hierarchy.**

Chelonii Latreille, 1800.

Cryptodira Cope, 1868.

Chelonioidea Oppel, 1811.

Dermochelyoidae Baur, 1888.

*Ocepechelon* gen. nov.

**Figure 2:**
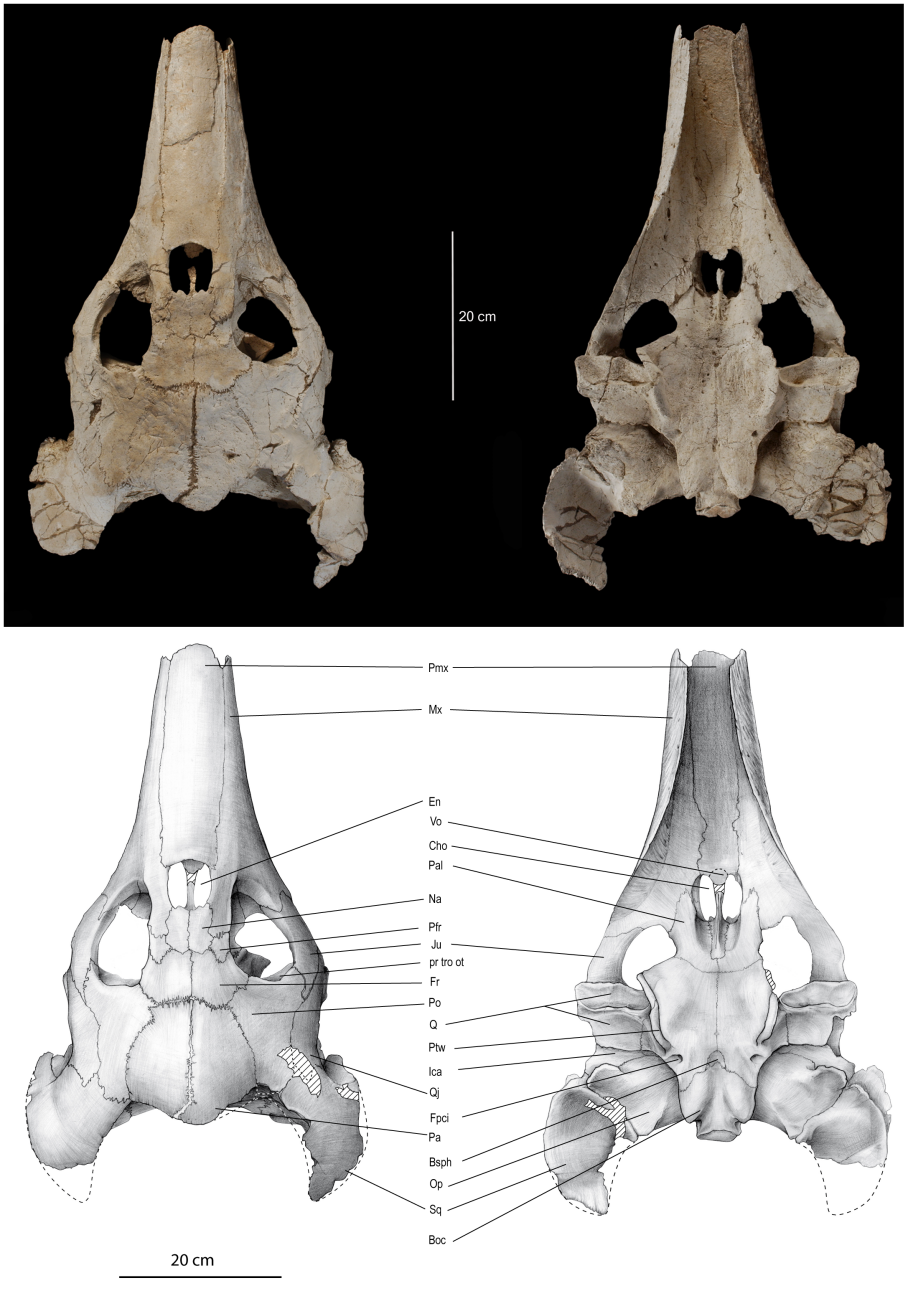
*Ocepechelon bouyai* gen. et sp. nov., dorsal (left) and ventral (right) views of the skull. OCP DEK/GE 516, Holotype, Phosphates (Upper Level III, Upper Maastrichtian, uppermost Cretaceous), Sidi Chennane area (Oulad Abdoun Basin, Morocco). Photographs and interpretative drawings. **Abbreviations**: Boc, basioccipital; Bsph, basisphenoid; Cho, choanae; En, external nare; Fpci, *foramen posterior canalis carotici interni*; Fr, frontal; Ica, *incisura columellae auris*; Ju, jugal; Mx, maxilla; Na, nasal; Op, opisthotic; Pa, parietal; Pal, palatine; Pfr, prefrontal; Pmx, premaxilla; Po, postorbital; pr tro ot, *processus trochlearis oticus*; Pt, pterygoid; Ptw, pterygoid wing; Q, quadrate; Qj, quadratojugal; Ra, marks of the rhamphotheca; Sq, squamosal; Vo, vomer. Scale = 20 cm.

**Figure 3:**
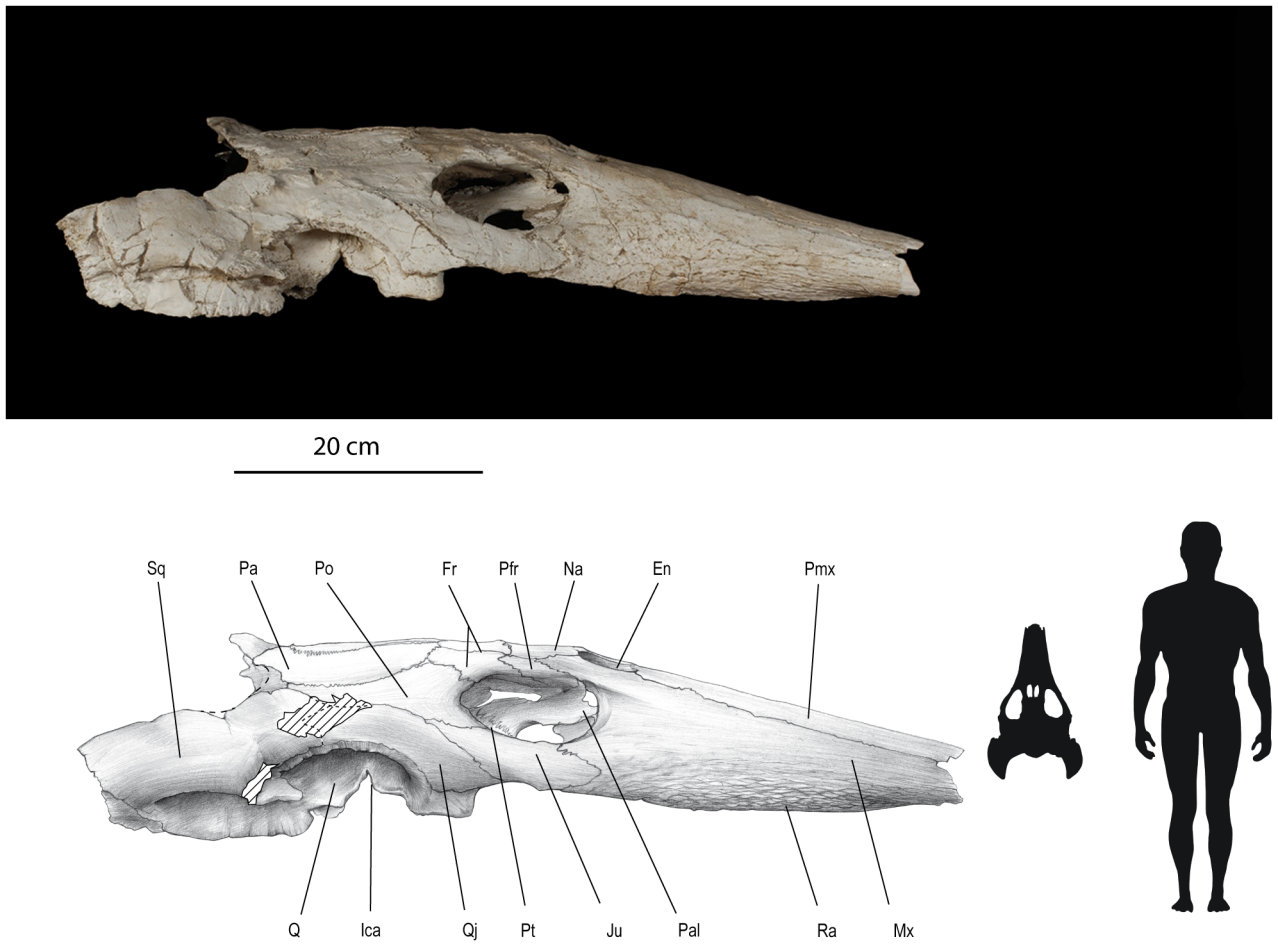
*Ocepechelon bouyai* gen. et sp. nov., right lateral view of the skull. OCP DEK/GE 516, Holotype, Phosphates (Upper Level III, Upper Maastrichtian, uppermost Cretaceous), Sidi Chennane area (Oulad Abdoun Basin, Morocco). Photograph and interpretative drawing. **Abbreviations**: En, external nare; Fr, frontal; Ica, *incisura columellae auris*; Ju, jugal; Mx, maxilla; Na, nasal; Pa, parietal; Pal, palatine; Pfr, prefrontal; Pmx, premaxilla; Po, postorbital; Pt, pterygoid; Q, quadrate; Qj, quadratojugal; Ra, marks of the rhamphotheca; Sq, squamosal. Scale = 20 cm.

**Figure 4:**
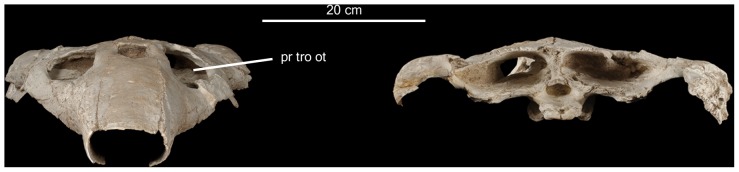
*Ocepechelon bouyai* gen. et sp. nov., anterior (left) and posterior (right) views of the skull. OCP DEK/GE 516, Holotype, Phosphates (Upper Level III, Upper Maastrichtian, uppermost Cretaceous), Sidi Chennane area (Oulad Abdoun Basin, Morocco). Photographs. **Abbreviation**: pr tro ot, *processus trochlearis oticus*. Scale = 20 cm.

**Figure 5:**
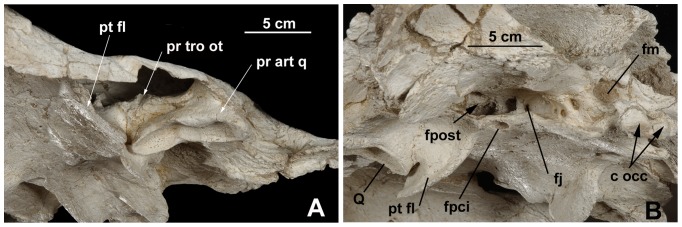
*Ocepechelon bouyai* gen. et sp. nov., details of the skull. OCP DEK/GE 516, Holotype, Phosphates (Upper Level III, Upper Maastrichtian, uppermost Cretaceous), Sidi Chennane area (Oulad Abdoun Basin, Morocco). **A**, lateroventral view of the skull (left side), showing the *processus trochlearis oticus* above the *processus articularis quadrati*. **B**, posteroventral view of the skull (left side) showing the widely opened *fenestra postotica*. **Abbreviations**: c occ, *condylus occipitalis*; fj, *foramen jugulare posterior*; fm, *foramen magnum*; fpci, *foramen posterior canalis carotici interni*; fpost, *fenestra postotica*; pr art q, *processus articularis quadrati*; pr tro ot, *processus trochelearis oticus*; pt fl, pterygoid flanges; Q, *processus articularis quadrati*. Scale = 5 cm.

**Figure 6:**
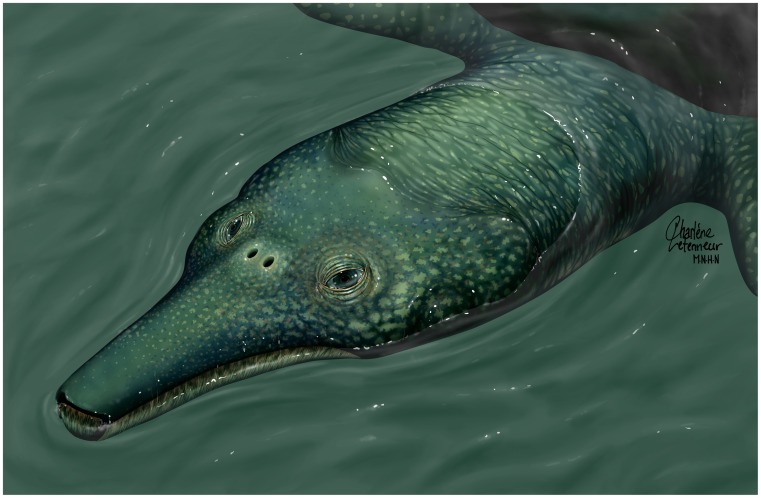
*Ocepechelon bouyai* gen. et sp. nov., life reconstruction. (Published with the permission of C. Letenneur/MNHN under a CC-BY license).

**Figure 7:**
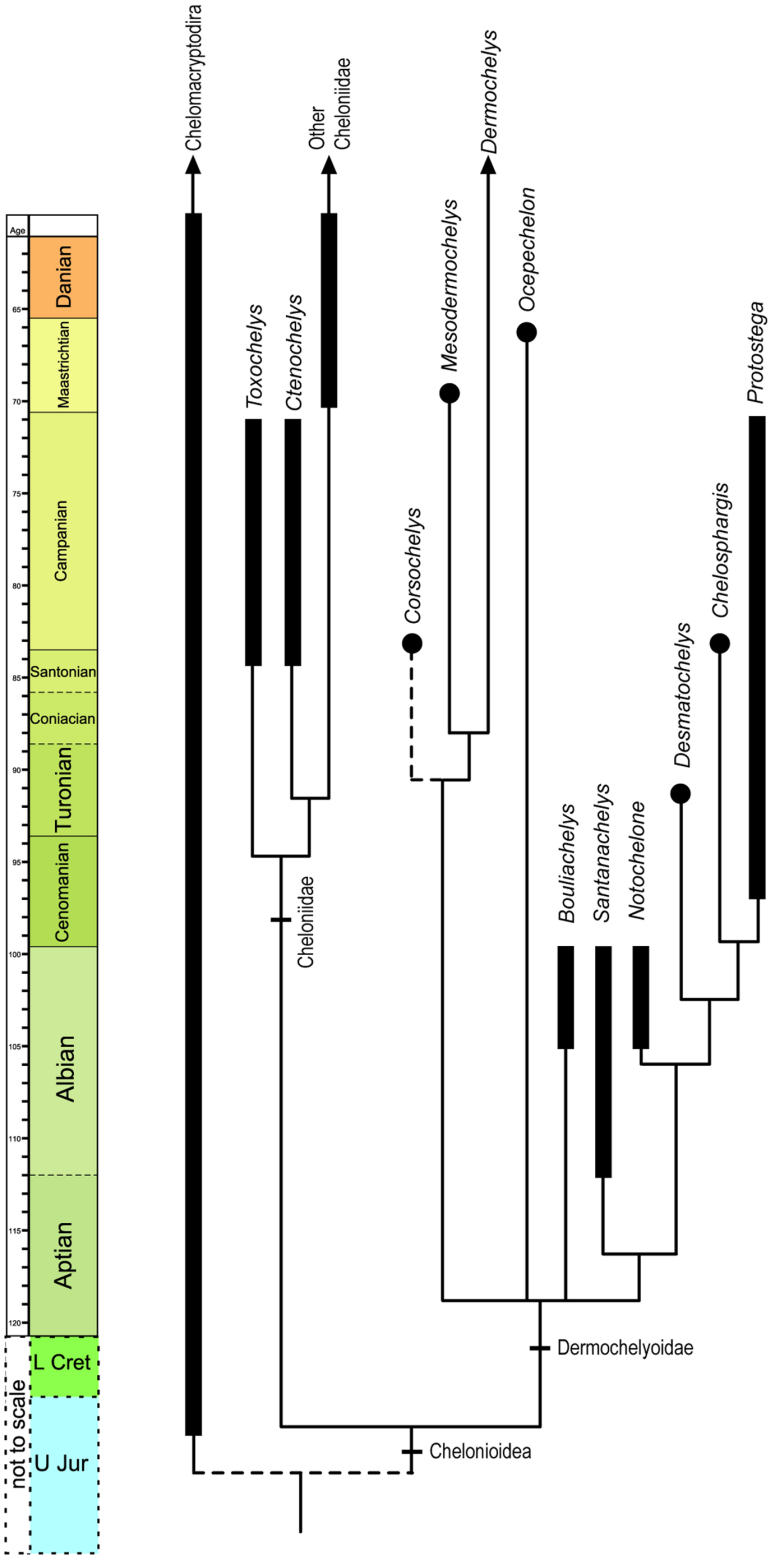
Chelonioidea time-calibrated consensus phylogenetic tree showing the phylogenetic relationships of *Ocepechelon* gen. et sp. nov. *Ocepechelon* is a remote relative of the extant leatherback turtle *Dermochelys coriacea* and of fossil protostegids.

*Ocepechelon bouyai* sp. nov.

(Figures 2–7, Figure S1)

#### ZooBank life science identifer (LSID) for genus

urn:lsid:zoobank.org:act:EB9E5777-6745-47A9-B293-0AAFBC0ED40F.

#### ZooBank life science identifer (LSID) for species

urn:lsid:zoobank.org:act:06CF16B2-E802-4DB4-87F6-DB061473E2DA.

#### Etymology

Genus name from OCP, acronym for the *Groupe Office Chérifien des Phosphates*, the mining company exploiting phosphatic deposits in Morocco, and from Χελώνη (chelone), meaning turtle in Greek; species name from Mr. Baâdi Bouya, engineer geologist, head of the OCP Geological Survey in Khouribga, for his help during our fieldwork.

#### Holotype

OCP DEK/GE 516, a complete skull found isolated, *Office Chérifien des Phosphates* collections, Khouribga (Morocco).

#### Type locality and horizon

Trench 2 (N32° 39, 516′; W 06° 38, 280′), Sidi Chennane area, SE part of the Oulad Abdoun Basin, Khouribga Province, Morocco (Figure 1A–B); Phosphatic deposits, base of the Upper Level III (ULIII), Upper Maastrichtian (uppermost Cretaceous) (Figure 1C) [3], [16].

#### Diagnosis

For genus and species, by monotypy. *Ocepechelon bouyai* gen. nov. sp. nov. differs from all other turtles in: a bony pipette-like snout consisting of an elongated arched rostrum with dorsoventrally compressed dermal bones ending anteriorly by a hemitubular and anteriorly directed snout opening; fused horizontal premaxillae and curved ventrolaterally maxillae, located in front of a flattened and wide skull table, that is posterolaterally elongated into large squamosal wings. Posterodorsally placed external nares, partly situated between the anterior part of the orbits and surimposed to the choanae. Flattened nasal cavity. External nare-vomer-choanae-palatine area curved and smooth up to the ventral maxillary margins that bear the marks of a rhamphotheca, ventrally forming thin ‘triturating’ surfaces, that constitute a structure appropriate for flow regulation rather than for prey capture or food processing. Large orbits facing obliquely, located close to the dorsal roof surface and superimposed to the *fossae temporalis inferiores*, without any bony floor; orbit immediately medial to the cheek emargination with a posterior margin at the vertical level of the anterior border of the well forward located *processus articularis quadrati*; vertically flattened jugal ventral to the orbit. Strongly developed vertical pterygoid flanges, located medial to the short *processus articularis quadrati*, and extending ventrally beyond this process. Extensive quadratojugal, quadrate and squamosal, stretched forward and backward, thus arranged horizontally and obliquely rather than vertically; low and prominent *processus trochlearis oticus* constituted by the quadrate without any prootic contribution, its borders delimited by right angles, forming a wide dorsal subrectangular and slightly concave facet. *Incisura columellae auris* ventrally facing in a wide semicircular notch of the *meatus quadrati*. Posterior extremity of the quadrate sutured with the squamosal at the horizontal level of the *condylus mandibularis*. Skull roof widened and flattened, inducing a wide cerebral cavity and a lateral wall of the *cavum cranii* bowed anteromedially; ascending epipterygoid also laterally directed and contacting the similarly laterally directed descending *processus inferior parietalis*; *foramen nervi trigemini* located at the extremity of a short canal anterolaterally issued from the *cavum cranii*. Ventrally elongated basicranium: pterygoids and basioccipital, enclosing the basisphenoid, extend far posterior to the *condylus mandibularis quadrati*, which bears a wide and deep biconcave articular facet; basioccipital tubercles elongated with a protuberant, wide and flattened biconvex *condylus occipitalis* located between the elongated and widened paroccipital processes of the opisthotic and the squamosal wings. Large size (holotype skull 70 cm long and 43 cm wide).

### Comparisons and Phylogenetic Relationships

A detailed description of *Ocepechelon bouyai* gen. et sp. nov. (Figures 2–5) is available in Text S1 of Supporting Information S1. A detailed phylogenetic analysis (Figure 7, Figure S1) is provided in Text S2 of Supporting Information S1. A life reconstruction is provided in Figure 6.

As a result, on the basis of its skull, *Ocepechelon* is clearly a cryptodire and not a pleurodire because of: 1) the presence of a *processus trochelaris oticus*, well visible in frontal view through the orbit, backward to the orbit which is fully open posteriorly; 2) a preserved epipterygoid contact; 3) a posterior extension of the pterygoid (Figures 2, 4, 5A).

Despite the presence of pterygoid flanges (here convergently acquired), *Ocepechelon* is definitively not a pleurodire because of all the above mentioned characters and because of the absence of a *processus trochlearis pterygoideus* laterally protruding into the inferior temporal fossa. Upward curled, this process forms in pleurodires a groove (the *sulcus palatino-pterygoideus*), dorsally opened and connecting the orbit to the inferior temporal fossa, laterally to the braincase and medially to the closed posterior wall of the orbit. In the absence of any posterior wall and groove, it is fully open in *Ocepechelon* up to the braincase.

It is a Chelonioidea because of the absence of *foramina praepalatina* and the presence of closely set *foramina anteriora canalium carotici interni* located in the *sella turcica*, anterior to the *dorsum sellae*; although relatively low and wide (possibly as a consequence of the autapomorphic skull flattening), the *dorsum sellae* is separated from the *sella turcica* by an oblique crest in conformity with the condition in Chelonioidea.

It is a stem Dermochelyoidae sensu [17] because of the absence of any ventral medial contact between the fully ventrally reduced jugal and the palatal elements (palatine and pterygoid) linked to a full reduction of the *foramen palatinum posterius* (as in protostegines and *Dermochelys*), and because of the vomer no longer contacting the pterygoids.

The *foramen palatinum posterius* is absent like in some cheloniids but, in these, this absence is due to the presence of a secondary palate and the contact jugal-pterygoid is preserved. The well-developed rodlike *rostrum basisphenoidale* raising off from the pterygoid floor is similar to that of advanced cheloniids.

*Ocepechelon* is not a protostegid because the pterygoid, although taking part into the articular process of the quadrate, is not lowered up to the mandibular condyle, and because of the posterior position of the posterior carotid foramen that is fully embedded in the pterygoid. The small and anteriorly rounded ventral basisphenoid is like that of protostegids, but differs in being located posteriorly to the *incisura columellae auris*. The short dorsal parietal-squamosal contact is similar to that of *Protostega* but the small parietal posterodorsal emargination is not deep. The *Ocepechelon* skull, lateroposteriorly prolonged by the squamosal wings, gives an erroneous aspect of a strong emargination, while this is barely larger than in *Dermochelys*, as shown by the short medial posterior protrusion of the parietals (covering the supraoccipital). Like in at least *Corsochelys* (undescribed structure in other protostegids), the wide *fenestra postotica* includes the *foramen jugulare posterius* and the hypoglossi nerve foramens, because the *recessus scalae tympani* is not closed posteriorly by any lateroventral extension of the exoccipital toward the basioccipital and the pterygoid (Figure 5B).

## Discussion

### Suction Feeding Adaptations of *Ocepechelon*

Despite the tremendous diversity in form and function of the turtle skulls [4], [18], [19], the skull of *Ocepechelon bouyai* is one of the most unusual. Although the lower jaw and hyoid apparatus of this species remain unknown, the diet and feeding mechanics of *Ocepechelon* can be inferred from the architecture of its large and highly specialized skull (see Text S1 of Supporting Information S1 and Figures 2–5) [20]. The flattened, streamlined shape of the cranium, combined with the dorsal position and orientation of the orbits and external nares, suggest that *Ocepechelon* was an epipelagic turtle hunting close to the sea surface, as for example crocodilians. Its marine behaviour is supported by both the sedimentological context of the type locality and the chelonioid skull anatomy. As a Dermochelyoidae, *Ocepechelon* much likely possessed limbs modified into flippers, allowing an active swimming ecology in open-sea. Possibly referable postcranial material found in the same Maastrichtian Level III of the Oulad Abdoun Basin further supports this anatomical deduction (see Text S1, p 11–13, in Supporting Information S1).

The most intriguing feature of *Ocepechelon* is its pipette-like longirostrine bony snout. The thin, tubular snout of *Ocepechelon* was embedded ventro-laterally in a deep and non-triturating rhamphotheca, as indicated by an area covered by nutritive foramina and short longitudinal sulci. But the absence of cutting, shearing or triturating structures in the upper jaw precludes any use for direct capture or processing of food items.

*Ocepechelon* has a large head, an elongated upper jaw without food triturating structures, and a mouth aperture in a plan perpendicular to the forward movement, all morphological features characteristic of ram feeding tetrapods [21]. Nevertheless, ram feeders tend to have an oral orifice with a very large cross-sectional area. The snout of *Ocepechelon*, ending with a relatively small rounded forward-facing-mouth aperture, is reminiscent of the rostrum and mandible of the much smaller syngnathiform fishes (seahorses, pipefishes, and allies), which represent efficient designs for suction feeding [22]–[26].

Suction feeding is frequent in aquatic vertebrates (chondrichthyans, osteichthyans, amphibians, aquatic turtles and marine mammals) [21], [25], [27]–[34]. The aspiration tube of suction feeders is variably formed by the snout, independently from the presence of teeth. It is documented in living short-snouted freshwater turtles such as *Chelus* and *Chelydra*, and among extant marine mammals, in the long-snouted sperm whale *Physeter* and beaked whales (Odonteceti, Ziphiidae), as well as in more or less short-snouted species such as various delphinoids and walruses. In all these groups, the buccal tunnel is at least partly made of soft tissues; examples include the mucosa issued from an external adductor muscle in the freshwater turtle *Chelus*, the *Mundplätte* (figures in [33]), or the large gums, and extensive lower lips that line the mouth opening in beaked whales (diagrams for species of *Mesoplodon*, figs. 2 and 3 in [24], figures (partly) and diagrams in [34], including fig. 3 for a generalized odontocete). Because the tubular rostrum of *Ocepechelon* is made of bone, it is therefore unique among marine tetrapods.

*Ocepechelon* differs significantly from other suction feeding turtles [18], in its unique cranial architecture. It particularly shares some interesting resemblances with beaked whales. *Ocepechelon* and most beaked whales are large animals, and, as in all other extant cetaceans and *Ocepechelon*, the nostrils of beaked whales are posterodorsally shifted (Figures 2, 3, 6). They also share similarities on their feeding apparatus: elongated jaws (without triturating surfaces in *Ocepechelon* and toothless in most extant beaked whales, except for one or two pairs of apical teeth modified in tusks in adult males) and a small gape [24]. The mouth gape of *Ocepechelon* can be inferred from the diameter of the tube (about 6 cm). It is worth emphasizing the disproportion between the tiny size of the mouth opening and the large size of the skull (70 cm long). The snout diameter predicts that *Ocepechelon* was a small-prey hunter (e.g., small fishes, cephalopods and jellyfishes). In pipefishes the snout length is inversely related to the mouth cross-section and is considered as an evolutionary advantage since it reduces the time to reach the prey [23]. In *Ocepechelon*, the elongated rostrum and the round and small mouth much likely increased the velocity of the water influx [22].

Suction feeding does not require strong biting and is often associated with weakly developed jaw adductor muscles, as seen in edentulous odontocetes. According to the different suction feeding processes, the different parts of the jaw adductor muscles are not equally developed, as seen with the developed mucosa in *Chelus* [33], [35]. Dominant muscles in *Ocepechelon* were attached to the pterygoids, as suggested by the conspicuous large ventral pterygoid flanges of these bones, comparable in development to those of pleurodiran Podocnemididae. In these turtles, the *pars ventralis* of the muscle *pterygoideus* (anterior part of the *Musculus adductor mandibulae internus*), inserted between the developed flanges, has a special function of protraction of the lower jaw, acting in synergy with the rectracting component of the external adductor muscle [35]. Situated above the hyoid apparatus, this muscle couple could also have acted in combination with the piston function of the hyoid and tongue in the swallowing process. By deduction, the same system might have worked convergently in *Ocepechelon.* However, the muscular bauplan is basically identical for swallowing process in turtles, such as it is figured in the sharpening feeder pleurodire *Podocnemis* [35] (fig. 11) (which possesses pterygoid flanges) and the crushing feeder cryptodire *Chelonia* [35] (fig. 10).

Moreover, in *Ocepechelon*, the squamosals are exceptionally developed into broad posterolateral wings, providing large surfaces for visceral muscle attachment [35], much likely for an important hyoid and tongue musculature, as in other living turtles such as the leatherback *Dermochelys* [35], [36] and notably the suction feeder *Chelus* [32], [33]. The hyoid apparatus and associated musculature are also remarkably developed in cetaceans, especially in suction feeding beaked whales (diagrams and figs. in [24], [27], [37]).

The remarkable and unique anatomy of *Ocepechelon*, including a unique bony pipette-like longirostrine snout, exceptionnaly large pterygoid flanges and posteriorly developed squamosal wings, suggests a feeding strategy that was later paralleled by several cetaceans, including the beaked whales [24], [25], [34]. We hypothesize that it fed, as the latter, by generating a fast, large-amplitude depression within its buccopharyngeal cavity. Given the narrow tubular shape of its upper jaw, *Ocepechelon* probably performed small-gape suction (with minimally opened mouth), thereby enhancing the suction force for attracting and ingesting the prey (Figures 2, 3, 6, Video S1). In general, a small and circular mouth opening generates greater negative intraoral pressure than a laterally open and larger mouth. A part of the marine mammals, excluding beaked whales, perform a circular mouth opening through shortening and widening of the rostrum and jaws [24], [33] (e.g. odontocete mandibular bluntness studied in details in Werth [34]).

In aquatic turtles, the expansion of the buccal and pharyngeal cavities plays an important role, since the highly distensible oesophagus may serve as a water reservoir and entrap prey until the closure of the mouth, before swallowing the prey and finally expelling the water, a behaviour that we propose for *Ocepechelon* [29]–[33], [38] (Video S1). Many living turtles, including marine forms such as *Dermochelys*, have keratinized buccopharyngeal papillae that allow both filtering water and entrapping prey [39]. Beaked whales also possess lingual or palatal horny papillae that may help retaining the prey against the palate [24]. *Ocepechelon* could have possessed mouth papillae.

### Conclusion

During the Mesozoic, large marine carnivorous reptiles such as ichthyopterygians, sauropterygians, mosasaurid squamates and crocodyliformes exhibited a wide range of feeding strategies based on their tooth morphologies, from benthic crushing feeders to huge open-sea generalist predators [40]. However, despite this considerable diversity in the Mesozoic marine reptile modes of predation [40], [41], as well as a broad overlap of their tooth morphospaces with those of Cenozoic marine mammals, indicating multiple feeding behavior convergences [42], suction feeding, though being a common feeding strategy in aquatic vertebrates [34] has been extremely rarely reported among Mesozoic marine reptiles. It has been hypothesized (but without any concrete argument) for *Hupesuchus*, a small marine reptile from the Middle Triassic of China [43] and recently postulated for *Shonisaurus* [44], [45] and *Shastasaurus* [46], both large toothless Triassic ichthyosaurs, interpreted as suction feeders also comparable to many extant odontocetes.

The unique anatomy and ecology of *Ocepechelon* suggest a prey capture behavior similar to that of several extant suction feeding odontocetes. Furthermore it is the only bony pipette-feeder ever reported among tetrapods. Irrespective of its feeding strategy, *Ocepechelon* dramatically illustrates the anatomical and taxonomic diversification of chelonioid turtles during the Late Cretaceous (Figures 6, 7). It combines turtle morphology with a feeding device that parallels the adaptation of modern pipefishes and some living odontocetes.

With its peculiar morphology, *Ocepechelon* likely occupied a different ecological niche compared to other coeval marine reptiles [3], [4], [8], [16]. Its specialization further emphasizes the tremendous diversity that prevailed among the fishes and reptiles in the shallow marine environments of the Late Maastrichian Phosphates of Morocco, shortly before the K/Pg mass extinction event [47], [48].

## Supporting Information

Supporting Information S1 **Detailed description, phylogenetical analysis, additional references and Figure S1.** Text S1: Detailed description. Text S2: Phylogenetical analysis (based on a slightly modified existing data matrix of morphological characters) including the list of characters, their discussion, the matrix and the details of the analysis with bootstrap values. Additional references: for detailed description and phylogenetical analysis. Figure S1: Chosen examples of cladograms obtained from the modified Kear & Lee (2006) matrix [15]; S1.1, removing outgroups one after the other, except the hypothetical taxon (cladograms not presented here display only minor changes compared to the ones illustrated); S1.2, Additional tree tested from the matrix of Figure S1.1 (C1 and C2), with the hypothetic taxon and Chelomacryptodira as outgroups; As in the strict consensus (Figure S1.1–C1), L = 207, Ci = 54, Ri = 62.
(DOC)

Video S1 **Animated 3D reconstruction of the suction feeding process in** ***Ocepechelon*** **(Maxon Cinema 4D).**
(MP4)
