# Correction: A Giant Chelonioid Turtle from the Late Cretaceous of Morocco with a Suction Feeding Apparatus Unique among Tetrapods

**DOI:** 10.1371/annotation/b4b475f1-6bcc-424a-87dc-aa5fabcc9288

**Published:** 2013-07-15

**Authors:** Nathalie Bardet, Nour-Eddine Jalil, France de Lapparent de Broin, Damien Germain, Olivier Lambert, Mbarek Amaghzaz

There was an error in affiliations for the first, third and fourth authors. The correct affiliation is: 1. CR2P, UMR 7207 CNRS-MNHN-UPMC, Département Histoire de la Terre, Muséum National d’Histoire Naturelle, Paris, France. 

