# Supplementary material for: A Giant Chelonioid Turtle from the Late Cretaceous of Morocco with a Suction Feeding Apparatus Unique among Tetrapods
Source: PLoS One. 2013 Jul 11;8(7):e63586. doi: 10.1371/journal.pone.0063586 (PMC3708935; doi:10.1371/journal.pone.0063586)
Supplement: Supporting Information S1 — Detailed description, phylogenetical analysis, additional references and Figure S1. Text S1: Detailed description. Text S2: Phylogenetical analysis (based on a slightly modified existing data matrix of morphological characters) including the list of characters, their discussion, the matrix and the details of the analysis with bootstrap values. Additional references: for detailed description and phylogenetical analysis. Figure S1: Chosen examples of cladograms obtained from the modified Kear & Lee (2006) matrix [15]; S1.1, removing outgroups one after the other, except the hypothetical taxon (cladograms not presented here display only minor changes compared to the ones illustrated); S1.2, Additional tree tested from the matrix of Figure S1.1 (C1 and C2), with the hypothetic taxon and Chelomacryptodira as outgroups; As in the strict consensus (Figure S1.1–C1), L = 207, Ci = 54, Ri = 62. (DOC) [file pone.0063586.s001.doc]

Bardet et al – Supporting Information – S1

Text S1 – Detailed description (Figures 2-6) p. 1-13

1. **Skull description ………………………………………………………………….. p. 2-10**
   1. **General and dorsal views**
   2. **Lateral view**
   3. **Medial view**
   4. **Ventral view**
2. **Lower jaw morphology hypothesis …………………………………………….. p. 10-11**
3. **Potential postcranial material and size hypothesis …………………………… p. 11-13**

Text S2 - Phylogenetical analysis (Figure 7, Figure S1) p. 14-48

1. **Considerations about the included taxa ……………………………………….. p. 14-17**
   1. **Problems of the Outgroups**
   2. **Problems of the Ingroup**
2. **Character definition and polarization …………………………………………. p. 17-19**
   1. **Outgroups**
   2. **Ingroup**
3. **Results ………………………………………………………………………….... p. 19-31**

**Figure S1.1.A**

**Figure S1.1.B**

**Figure S1.1.C**

**Figure S1.2**

1. **Character list ……………………………………………………………………. p. 32-45**
2. **Character matrix ………………………………………………………………... p. 46-48**

**Additional references p. 49-54**

**Figure S1 p. 55-56**

Text S1 – Detailed description (Figures 2-6)

*Ocepechelon bouyai* gen. et sp. nov. is one of the best records of the rare cryptodiran turtles, known from the Cretaceous of Africa, which includes chelonioids from the Maastrichtian Phosphates of Morocco (see Table 1), the Maastrichtian of the Western Desert of Egypt, and a sandownid from the Turonian of Angola (Bardet et al., 2010; Bardet et al., in press; Lapparent de Broin, 2000; Lapparent de Broin & Werner, 1998; Mateus et al., 2009; Tong & Meylan, 2013).

**1. Skull description**

The skull of *Ocepechelon* is unusual in its longirostrine tube-like snout, much likely constituting a tool adapted to pipette-suction feeding. Related to the development of this rostral tube, the odd bony external nare is located posteriorly and dorsally, in the interorbital region. It overhangs the choanae in an original way, without a secondary palate and without any nasal tract connecting the internal and external nare. The dorsal position of the nare, close to the skull roof surface, and its dorsal orientation suggest that *Ocepechelon* was most probably an epipelagic suction feeder probably hunting close to the sea surface. The skull elongation affects not only the longirostrine preorbital part of the skull of *Ocepechelon*, but also its postorbital area, with very expanded and lateroposteriorly flared squamosals, giving together with the elongated snout a general triangular aspect to the skull in dorsal view. According to the skull size and shape, and on the basis of comparisons with skull/shell proportions in the largest protostegids *Archelon* and *Protostega* (Hay, 1908; Wieland, 1909; Zangerl, 1953a), *Ocepechelon* could have reached a body length of at least 240 cm, from snout to tail end (see below).

**1.1. General and dorsal views**

The snout is lightly ossified and, except the arched maxillae, the snout elements (premaxillae, palatine, vomer and nasal area) are strongly compressed. On the internal and external sides of the maxillae a longitudinal line delimits the area that was covered by a high horny rhamphotheca. This area is covered by nutritive foramina prolonged by fine and short longitudinal sulci. The dorsal surface of the snout, made by elongated and fused premaxillae, is smooth without any indication of the presence of a horny beak anteriorly. The skull surface is smooth without any scute sulci. The skull of *Ocepechelon* was probably devoid of scutes and was likely covered by skin, as in *Dermochelys*, Trionychids and probably advanced protostegids.

The orbits are large, facing dorsolaterally, slightly inclined; they are close to the dorsal surface of the skull roof. They overhang and are confluent with the *fossa temporalis inferior*, which, in the absence of orbit floor, slightly modify the insertion surface and the course of a part of the adductor muscles. Thus, the bony insertion place for the dorsal pars of the *M. pterygoideus* (that is an anterior portion of the *M. adductor mandibulae internus*), rising in cryptodires from the orbit base surface, is here reduced. However, the ventral pars of the *M. pterygoideus*, running below the pterygoids as in extant turtles (Schumacher, 1973), was much more developed here, according to the important pterygoid ventral flanges, that parallelize the large pterygoid wings of pleurodiran Podocnemididae. This condition also possibly modify the space and course of some muscle mass, such as the *M. adductor mandibulae externus* (rising in turtles from the *fossa temporalis inferior*), that is housed in the *fossa temporalis superior* (Schumacher, 1973), inside the presently wide and long but flattened skull table. However, the pterygoid muscle bauplan is identical in Pleurodires with more or less important pterygoid flanges - such as it is figured in the sharping feeder *Podocnemis* (Schumacher, 1973, fig. 11), and, in Cryptodira, - such as it is figured in *Chelonia* (Schumacher, 1973, fig.10) a powerful crushing feeder; these muscles have a role in prey swallowing. The eyes were smaller than could be expected from the orbits as they were originally surrounded by muscles and by probable salt glands in the posterior orbit part. Indeed, primitive lagoonal turtles and marine chelonioids always possess large orbits (Gaffney, 1975a, b, 1979; Hirayama, 1995; Jalil et al, 2009; Schumacher, 1973). Thus, in the back of the orbit, the *processus inferior parietalis* is narrow and dorsally notched in marine extant Cheloniidae, the lagunar Jurassic *Solnhofia* aff. *parsonsi* from Garissou (Lapparent de Broin et al., 1996; Lapparent de Broin, pers. obs.) and the lagunar protostegid *Santanachelys* (Hirayama, 1998), to host the posterior part of the large salt glands (Hirayama, 1998; Renous et al., 2008; Schumacher, 1973). There is no notch in the externally directed parietal descending processes of *Ocepechelon* but the presence of a salt gland is however possible.

The external nare is behind the premaxillary rectangular plate. Compared to related forms, it is remarkably shifted backward and dorsally, up to and partly between the orbits. Otherwise, dorsal nare is only known in the protostegine *Archelon*, although slightly inclined and not shifted backward. In *Ocepechelon*, the nare is directly superimposed to the choanae, separated only by a very low height due to the dorsoventral snout compression. The nasals are present in the interorbital area, behind the external nare. They are rectangular and meet sagittally. Two small anterior processes project from the anterior border of the nasal and overhang the external nare. Between the nare and the anterior half of the orbits, the maxillae send small posterodorsal processes, which frame the anterior part of the nasals and overlap the anterior part of the vertically descending processes of the prefontals. Both small prefontals appear dorsally between the maxilla anteriorly and the frontal posteriorly, on the side of the posterior part of the nasal. Their descending process projects very slightly in the internal nare border but its contact with the vomer is not clear, being ventrally lost and possibly also dorsally (damaged not clean surface). Each frontal borders the orbit between the prefrontal and the postorbital, which are therefore separated from each other. This interorbital configuration is not frequent in turtles. It is similar to the condition of the protostegid *Desmatochelys*, differing only in the relative extension of interorbital bones (Gaffney, 1979; Hirayama, 1995; Zangerl & Sloan, 1960).

The postortbitals are long and project posteriorly alongside the parietals, but they do not reach the posterior border, being excluded from it by a faint parietal-squamosal contact. The parietals are slightly pointed medioposterioly, covering a short *crista supraoccipitalis*, not visible in dorsal view: its posterior end is broken and slightly incomplete. As the *crista supraoccipitalis* is short, the posterodorsal emargination appears shallow between the posterior end of the parietal and the squamosal; it is slightly larger than in *Dermochelys*, slightly less deep than in extant Cheloniidae and contrasting with the very large emargination of some cryptodire marine turtles, including protostegids such as *Desmatochelys*, *Protostega*, and *Archelon* (Hay, 1908; Hirayama, 1995; Wieland, 1909). Nevertheless, the skull roof of *Ocepechelon* is greatly laterally widened by the posterolateral extension of the squamosal, forming a strong posterior concavity, which is however not equivalent to the posterior emargination seen in these last turtles.

**1.2. Lateral view**

Behind the tubular snout, the maxilla, the jugal, and the quadratojugal delimit a faint lateral emargination, just below the orbit. The jugal is located below the orbit, extending slightly behind the posterior orbital margin. It is a flattened bony rod without any median projection or any contact with the palatal elements. In lateral view, the jugal projects slightly anteriorly into the maxilla; posteriorly, it is bevelled between the postorbital and the quadratojugal. The quadratojugal is a stretched flat bone covering in a wide arch the dorsolateral head of the quadrate. Almost the entire lateral surface of the quadrate forms the extended, deep, and kidney-shaped *cavum tympani*. Midway along its ventral border is the narrow groove notch of the *incisura columellae auris*, for the passage of the *columella auris*. The middle ear area is upsets backward, from the quadratojugal to the squamosal, compared to other marine turtles where the area is more vertical: here, the quadrate is extended forward and backward and results very low; the *processus articularis* is short and pushed forward with the *processus trochlearis oticus*; posteriorly, the quadrate extends well beyond the *processus articularis*, and it is so much ventrally bended that the *incisura columellae auris* groove opens into a wide half circular space; consequently, the *incisura* faces ventrally rather than posteriorly or postero-ventrally as it faces in other marine turtles; besides, the posterior border of the quadrate in contact with the squamosal comes at the horizontal level of the *condylus mandibularis*. This extreme modification of the quadrate is unique to *Ocepechelon*. In marine cryptodire turtles, the quadrate is more vertical and the angle of the i*ncisura* is relatively wide primitively, and postero-ventrally directed; in the protostegine *Archelon ischyros* and *Microstega copei*, the quadrate *incisura* angle is wide, but to a lesser degree than in *Ocepechelon*, and it is directed posteriorly; only *Archelon* has a quadrate that is slightly upset forward but not stretched.

**1.3. Medial view**

The quadrate of *Ocepechelon* is so upset that the forward displaced *processus trochlearis oticus* lies just above the *processus articularis* which is short in relation to other marine turtles*.* The *processus trochlearis* is constituted by the quadrate alone without any prootic contribution; it is stout and low, well delimited by angles and forming a wide dorsal subrectangular and slightly concave facet. It is well visible in the background of the orbit in frontal view (Figure 4, left), just below the posterior orbit margin in dorsal view (Figure 2, left) and above the *processus articularis quadrati* in ventral view (Figure 5A).

The skull roof is wide and low. The cerebral cavity is similarly wide and low. The lateral wall of the *cavum cranii* is anteriorly bowed; the *processus inferior parietalis* is a wide anteroposteriorly directed parasagittal plate. Laterally to the *sulcus cavernosus*, its anterior part is bowed dorsolaterally and externally up to its contact with the epipterygoid, in front of the prootic. The epipterygoid is a rod-like bone erected dorsally above the pterygoid, close medially to the palatine-pterygoid suture. It is exteriorly directed, ventrodorsally toward the similarly exteriorly directed *processus inferior parietalis*. The trigeminal nerve V exits from the *cavum cranii* through the *foramen trigemini*, at the end of a short canal anterolaterally directed between the *processus inferior parietali*, the epipterygoid, and the pterygoid. Medially to the sulcus, the *dorsum sellae* is wide and relatively low, related to the wide cerebral cavity and the skull flattening. It delimits posteriorly the *sella turcica*, making a short vertical wall with a sagittal crest separating the anterior foramina of the inner carotid; forwards, the trabeculae extend on each side of the *sella tucica* and unit to form a developed rodlike *rostrum basisphenoidale*, oval in section, which raises off the pterygoid floor as in advanced cheloniids. Laterally, the suture with the prootic runs upward posteriorly, so that the anteroventral part of the *processus inferior parietalis* is triangular.

**1.4. Ventral view**

The pipette-like longirostrine snout is lightly built and lacks functional triturating surfaces as well as a secondary palate. The *foramen praepalatinum* and the *foramen palatinum posterius* are also absent. The choanae are separated by the vomer narrov pillar and lie just below the external nare, which displays a similar shape. The nasal cavity has a short “subrectangular box” shape, completely unusual among turtles. The vomer is an elongated bone ending anteriorly in a small, rounded, and flat area, anchored medially in the fused premaxillae. In its medial part separating the choanae, the vomer is much narrowed, bearing a thin and vertical short crest; posterior to the choanae, the vomer extends between the palatines in a narrow and long strip along the half length of palatines; its ventral sagittal crest weakens posteriorly; there is no contact with the pterygoids. A very small palatine lateral process is present at the contact with the maxilla (better preserved on the right side), but there is no lateral process of the pterygoid. When developed, the lateral pterygoid cryptodiran process acts as a guide for the lower jaw during the adduction of the lower jaw. The palatines meet medially for a short distance, being anteriorly separated from each other by the vomer. They do not reach either the fused premaxillae or the vomer anteriorly, nor the jugal laterally, and are gently incurved on each side of the choanae. They are narrowed just in front of the suture with the pterygoids. The two pterygoids meet medially. They are unusual in possessing well developed ventral vertical flanges located medially to the quadrate processes and extending from the level of the mid part of the *fossae temporalis inferior* to the level of the *incisura columellae auris*. These ventral flanges or wings (deformed and folded down on the right side, slightly anteriorly incomplete on left side) with rounded ventral borders, pass just between the *processus articularis quadrati*. The presence of such developed ventral flanges on the pterygoids is unusual in cryptodires; these flanges appear as homologous to the pterygoid wings of Eupleurodira.

The pleurodiran pterygoid wings are medial, posterior to, and independant from the *processus trochlearis pterygoideus* which projects laterally in the *fossa inferior temporalis*; the process margin curves upwards, and narrows to anteriorly extend, constituting an orbitopterygoid groove (Schumacher, 1973), the *sulcus palatino-pteygoideus*, dorsally opened and anteriorly ending in the back of the orbit. This gives a vertical cavity located laterally to that of the *cavum cranii* and laterally bordered by a posterior orbit wall, both structures visible in frontal view. In *Ocepechelon*, the *processus trochearis* is made by the otic (quadrate/prootic) area and not the pterygoid: the back of the orbit is fully open laterally on each side of the *cavum cranii* up to the cheek wall, and, in frontal view, the *processus trochlearis oticus* is visible in the background (Figure 4) like in other non pleurodiran turtles.

In pleurodires, the pterygoid wings extend from the palatine anteriorly toward the back at the level of the *processus articularis quadrati* and are generally slightly developed, except in Podocnemididae. In these, they posteriorly extend medially to cover most part of the podocnemidid fossa (*fossa pterygoidea* or muscular chamber) which is sunk in the surface of the pterygoid posteriorly, the quadrate medially, as well as the prootic and the basisphenoid lateral border. In pleurodires, the space between the wings is filled by the pterygoid muscle (anterior part of the *adductor mandibulae internus* (Schumacher, 1973). In Podocnemididae (with strong pterygoid wings as in *Ocepechelon*), the most anterior pterygoid muscle parts arise from laterally and anteriorly on the trochlear pterygoid process border and orbitopterygoid groove in the temporal fossa, running medioanteriorly; a posterior pars arises from the deep podocnemidid fossa, also running medioanteriorly (Schumacher, 1973, fig. 11). In *Ocepechelon*, the pterygoid flanges extend far ventrally and posteriorly, beyond the level of the quadrates, due to the forward position of the *processus articularis quadrati*. As supposed in *Ocepechelon*, like in extant cheloniids (Schumacher, 1973, fig. 10), the pterygoid area was filled by the ventral pterygoid muscle pars; in extant taxa, it arises from the palatine-anterior pterygoid area and runs posterolaterally toward the dorsal lower jaw end. However, the muscle ought to be more developed in *Ocepechelon* than in cheloniids, according to the great development of the flanges.

The *condylus mandibularis quadrati* of *Ocepechelon* has a very wide articular surface with the mandible; it shows two concavities separated by a median faint prominence, as in extant cheloniids, bordered by acute margins: the deep biconcavity indicates the presence of cartilage during life (Figure 2 right, Figure 5A). Posteriorly to the *processus articularis quadrati*, a prominent crest borders anteroventrally the groove (*incisura*) for the *columella auris*, as in other chelonioids, but here the *incisura* faces ventrally instead of posteroventrally or posteriorly. This crest was originally covered by connective tissue, probably extended until the strong lateroanterior crest of the *processus paroccipitalis opisthotici*; the tissue ventrally wrapped the Eustachian tube, passing below the *columella* towards the *meatus quadrati* at the external border. The pterygoid and the basioccipital are more extended behind the *processus articularis quadrati* than usually seen in chelonioids; they surround a very small and anteriorly rounded basisphenoid; this is fully posterior to the *incisura columellae auris*, related to the forward inclination of the quadrate articular process. The basioccipital has an extensive contact with the pterygoid, by the way of an anteroposterioly directed suture. At midway between this suture and the *columella*, is located the large and rounded *foramen posterius canalis carotici interni*, ventroposteriorly in the pterygoid, anterior and separated from the margin of the pterygoid (fully embedded in the bone), like in marine chelonioid turtles (Figure 5B) except protostegids. Given the course of the inner carotid toward the *sella turcica* in the basisphenoid, the position of the *foramen posterius canalis carotici internii* at the level of the ventral basisphenoid suggests that the basiphenoid is much larger dorsally, being ventrally covered by the pterygoid. This is confirmed when looking in the cerebral cavity. The posterior tubercles of the basioccipital are well developed in length and width and oriented posteroventrally, on each side of the base of the protuberant, wide, and dorsoventrally flattened *condylus occipitalis*; the exoccipitals are fused dorsomedially, above the basioccipital, which is extended below in a transversal, wide and convexe condylian band. Such a condyle favours dorso-ventral movements of the head, more than lateral movements. This is consistent for a cryptodiran turtle with a neck retracting in a vertical plane. The posterior oblique border of the pterygoid contributes to delimit the external border of the *fenestra postotica* (lateroposterior opening of the *cavum acustico jugulare*), which is hidden in ventral view.

The squamosal has an extensive contact with the *processus paroccipitalis* of the opisthotic and it is much extended posteriorly. Their combined surface, anteriorly delimited by the strong and high crest of the paroccipital process, is bumpy at opisthotic and much concave at squamosal. This is the ventral insertion area for the abductor muscle, the *depressor mandibulae* that extends between the posterior end of the squamosal and the back of the lower jaw (Schumacher, 1973). Always well developed in extant cheloniids (Gaffney, 1979; Lapparent de Broin et al., 2007; Schumacher, 1973), the surface is much more developed here, possibly related to the elongation of the snout. As a suction feeder (see main text), *Ocepechelon* probably did not need a strong musculature for a small mouth opening. Insertion surfaces of muscles are developed on the lateral part of the flared squamosals: the wide squamosal expansion provides important surfaces for the insertion of visceral musculature (*constrictor colli, digastricus*), which must have played, with other hyoid and tongue muscles, an important role in suction feeding and swallowing processes. This feeding hypothesis is further supported by the reduction of space for a part of the adductor mandibular musculature, as well as the moderate development of the insertion surfaces for the anterior adductor musculature in the orbital cavity, on the short supraoccipital process, and possibly in the *fossa temporalis superior*. However, if the fossa is relatively low, the skull table is wide and provides enough surfaces at least for a moderate adductor musculature; the trochlear process of the quadrate is developed in width for that reason. The very short and wide anterior surface of the quadrate (Figure 5A) could give rise to the medial part of the muscle *adductor externus mandibulae* (see main Text) and the muscle *adductor mandibulae posterior* like in other turtles (with eventually the prootic and parietal) (Schumacher, 1973). Nevertheless, one must note that the extreme horizontal inclination of the quadrate results in a more oblique orientation of the jaw adductor musculature. The dorsal temporal musculature was not as much developed as in turtles with strong emarginations, such as *Chelus* or *Chelodina* and many others (Lemell et al., 2010; Van Damme & Aerts, 1997; Gaffney, 1979). The *crista supraoccipitalis* is short and covered by the parietals medially. In various turtles, the *crista supraoccipitalis* is a posteriorly projecting spine or vertical plate (cheloniids) that increases significantly the attachment area for a part of the adductor musculature. Here, the *crista supraoccipitalis* is short and covered by the parietals medially, but the inner supraoccipital surface is wide. However, with a short *crista suproccipitalis*, the insertion area for the adductor muscles is limited. Powerful adductors for bite are not necessary in the case of *Dermochelys* (feeding on soft prey, mostly jellyfish), probably no more than in *Ocepechelon*, that was unable of prehension mechanism and considered as a specialized suction feeder (see main text). In any case, the unusual architecture of the skull of *Ocepechelon* allows a specific musculature organisation, different from that of other turtles and favourable to suction and swallowing.

The posterior emargination of the parietals on each side of the crest is short. The strong expansion of the squamosal wings gives the impression of a strongly emarginated skull; however, this condition is not homologous to the dorsal temporal emargination of other turtles and is unique to *Ocepechelon.* The weak temporal emargination in *Ocepchelon* represents a primitive condition which can be reversed; it is rather similar to that of *Dermochelys*, even a little bit more pronounced, like in advanced cheloniids.

Inferoposteriorly, the *cavum acustico-jugulare* is visible, widely opened laterally and posteriorly in a wide *fenestra postotica*: the *recessus scale tympani* is not closed posteriorly by any lateroventral extension of the exoccipital toward the basioccipital and the pterygoid, as it is in Cheloniidae and *Dermochelys*, so that the *foramen jugulare posterius* for the Vagus nerve X, the accessory nerve XI, the *vena cerebralis posterior*, and the foramina for the hypoglossi nerves XII are all included in the limits of the *fenestra postotica* (Figure 5B). It is unclear if this primitive condition exists in protostegids where the fossa is undescribed; the condition is known and shared at least by *Corsochelys* and *Ocepechelon*, contrasting with Cheloniidae and *Dermochelys*.

**2. Lower jaw morphology hypothesis**

Based on the skull anatomy and comparisons with Protostegidae and extant Cheloniidae, hypotheses on lower jaw morphology can be infered. *Ocepechelon* much likely had a long jaw symphysis, which would have completed the rostral tube in a pipette-like structure; it extended from the oral cavity up to the anterior border of the choanae, where it split into two posterior branches. Indeed, in all turtles, the choanae are always located just posterior to the mandibular symphysis; therefore, their position may indicate the length of the symphysis and the position of the tongue behind. The unusual posterior position of the external nare of *Ocepechelon* has to be related to the development of the pipette-like snout adapted to suction feeding; in the absence of a secondary palate, the choanae had to be drawn back and their position indicates the posterior extremity of the symphysis. The symphysis was likely covered by a horny beak, corresponding to the maxillae rhamphotheca; the long mandible rhamphotheca had to be posterolaterally limited from the front of the coronoid process downwards the back of the jaw, as usual.

Assuming from the skull anatomy that the lower jaw of *Ocepechelon* was narrower than the upper jaw, as in all other turtles, the symphysis was necessarily narrow, and high enough to reach the dorsal limit of the maxillary ramphoteca; it was probably stout, higher than wide, and rectangular in section. It should have been elevated anteriorly into a spatula, in a way to close the circular mouth opening. The width of each posterior branch was inevitably inferior to the width of the *processus articularis quadrati* and each branch had the length comprised between the symphysis and the *processus articularis*. The *processus coronoideus* was necessarily low, according to the space available below the orbits, as in advanced prostostegids and differing from extant marine turtles (Hirayama, 1995). The *processus retroarticularis* had to be limited in length according to the possible place given by the lower jaw rotation during the mouth opening, more or less like in cheloniids, *Microstega*, and *Archelon*. However, because the quadrate is forward stretched, the articular facet was less inclined than in these other turtles. The rounded and inward curved maxillae imply a narrower mandible with a vertical lateral surface. The space between the upper and lower jaws could have allowed the expulsion of water after the prey aspiration.

**3. Potential postcranial material and size hypothesis**

It is difficult to infer the size of a turtle from its skull alone since the skull/shell proportion varies among turtles. However, the closely related and contemporaneous giant protostegids from the Senonian of North America (Hay, 1908; Hooks, 1998; Wieland, 1909; Zangerl, 1953a) may give a model for an approximation of the size of *Ocepechelon*. Because *Ocepechelon* is a Dermochelyoidae chelonioid on the basis of its skull anatomy, it is expected to be a marine turtle with large developed flippers and more or less lightened shell, comparable to protostegids.

The protostegids *Archelon* and *Protostega* have wide and rather rounded shells and large squared plastra in relation with their length with moderately long skulls: they are not longirostrine, so the skull width is probably a better parameter of comparison than its length. The length of the humerus could eventually give a better model, being less correlated with the shell shape. We have thus compared the postcranial elements known in the Maastrichtian Phosphates of Morocco to the known protostegids.

The Maastrichtian Phosphates of the Oulad Abdoun Basin have yielded several very large Chelonioid elements (OCP collection): dorsal shells with large pleural disc fontanelles, widely U-notched and and andindented nuchal, various star-shaped plastral elements with deeply indented edges, shoulder and pelvis (neither protostegid nor dermochelyid) elements, as well as humeri and femurs. They have been found at Grand Daoui (NE part of the Oulad Abdoun Basin), not far from Sidi Chennanne (locality of *Ocepechelon*, SE part of the Oulad Abdoun Basin), but in a slightly upper level of the same Upper Bed III. A similar indented plastral element probably originates from Grand Daoui (Tong & Hirayama, 2004). This plastral element was attributed to the Dermochelyidae (i.e. epifamily Dermochelyoidae, this work, sensu Bour & Dubois, 1986 corr. Gaffney & Meylan, 1988) by comparison with various Late Cretaceous chelonioids taxa; however, among them, *Protosphargis veronensis*, with deeply indented plastral elements, must be definitely attributed to the Cheloniidae, according to its large unique pelvic thyroid fenestra (Capellini, 1884; Cigala Fulgosi et al., 1980) and the dermochelyid s.s. *Corsochelys* referral is discussed (Zangerl, 1960; see character 27/1). As also shown in *Allopleuron* from Maastricht, now well figured (Mulder, 2003), the indented plastra are not exclusive to Protostegidae; besides, in these last ones, the hyo- and hypoplastra are more quadratic, less and differently indented, being more regularly arranged around each main corpus.

All these postcranial elements from Morocco may correspond to *Ocepechelon*, the only skull morphotype known in the Oulad Abdoun with a corresponding large size. These postcranial elements do not reach the size of *Archelon ischyros* (shell 193 cm long), but that of *Protostega dixie* (shell 154 cm long, skull 56.4 cm long). The largest shell from Grand Daoui (slightly incomplete) could have reached approximately 130 cm, but it does not represent the largest specimens, known from appendicular skeleton elements.

By comparison with these large protostegids, a carapace length of around 130-150 cm is expected for *Ocepechelon*, which could have reached 240 cm from snout to tail.

Two morphological types of humeri seem to be present in Grand Daoui, indicating the possible presence of two different large chelonioid taxa. None of them (respectively 53,2 cm; 52,3 cm; 59,7 cm) reaches the 65 cm of the humerus of *Protostega* *gigas*: the Grand Daoui largest humerus has the size of *Cratochelone* (60 cm), from the Albian of Australia (inferred maximum body length, without verification and possibly erroneous, approaching 4 m: Longman, 1915 in Kear & Lee, 2006).

The large humeri from Grand Daoui are typically ‘chelonioid’ but relatively derived and closer to *Rhinochelys* or *Desmatochelys* than to ‘Toxochelyidae’ sensu Zangerl (Hirayama, 1992); they have a much enlarged great trochanter in relation with the small trochanter and the head, a flat diaphysis and a flattened expanded distal part, posteriorly rounded and crossed by the radial nerve in the rounded ectepicondylar foramen. Reported to the same sagittal length, one specimen is relatively much wider, with a wider and longer great trochanter and a wider distal part, than three other ones; however, none is widened in the way of advanced protostegids (Hay, 1908; Wieland 1909) and the small trochanter is not as much modified in its position as seen in Dermochelyidae and advanced Cheloniidae and Protostegidae. It remains unclear if the large pelvis had a large thyroid fenestra, as in Cheloniidae, or two small thyroid fenestrae as in Protostegidae and Dermochelyidae.

As the skull, none of these postcranial elements can be surely referred to Protostegidae or Dermochelyidae and they could partly belong to *Ocepechelon*.

The large Late Cretaceous marine turtles are not the first large turtles recorded: large aquatic turtles have been found in the Jurassic (Gaillard et al., 2003 reported a shell up to 160 cm from the Kimmeridgian of Cerin, France). Others are recorded during the Early Cretaceous, including those of the Albian of Australia, as well as during the Late Cretaceous worldwide, and in different families, including *Allopleuron* from Maastricht. Large marine turtles will also appear during Tertiary times up to *Dermochelys*, the largest extant turtle.

Text S2 - Phylogenetical analysis (Figure 7, Figure S1)

Characters used are mostly those of Hirayama (1998, named 'H' along), with modifications of individual characters introduced by Kear & Lee (2006, named 'KL' along), and some new ones. Here we take advantage of the opportunity of the study of *Ocepechelon* *bouyai* gen. et sp. nov. to point out some problems related to the interrelationships of cryptodiran marine turtles, especially those concerning the definition and polarization of characters, and to propose some solutions.

As a whole, the H-KL matrix appeared to be the best matrix currently available for a complete study of marine turtles, including Mesozoic forms comparable to *Ocepechelon*, even it is not as well detailed as those found in studies on Cheloniidae (Hirayama, 1992, Brinkman et al., 2009; Parham & Fastovsky, 1997; Parham & Hutchison, 2003; Parham & Pyenson, 2010), Protostegidae (Hooks, 1998) and Dermochelyidae (Wood et al., 1996). Some important fossil taxa are not present in the KL matrix, such as *Rhinochelys* (a relatively poorly known protostegid) and *Allopleuron* (a Maastrichtian cheloniid), both previously included in Hirayama (1992, 1995) and Hirayama & Chitoku (1996). It should be noted that *Allopleuron* is now better known (Mulder, 2003); it is an interesting taxon exhibiting a derived cheloniid skull associated with a shell that seems partly homoplasic with that of some Dermochelyoidae. *Archelon* (a poorly known protostegid) is also lacking from the analysis. Finally, the Dermochelyidae family is poorly represented in the analysis, especially with the absence of Cenozoic taxa. The definition of the included taxa (the list of the included species or genera in the taxa groups) is basically taken from Gaffney & Meylan (1988), modified several times in later works, from Gaffney & Meylan (1988) to Gaffney et al. (1998), and from Hirayama (1985, 1992, 1995, 1997, 1998) and Hirayama & Chitoku (1996).

The KL matrix is sometimes difficult to use for two reasons:

**1. Considerations about the included taxa**

Even if references are given, it is not specified which genera and species are included in the larger taxa.

**1.1. Problems of the Outgroups**

a) Plesiochelyidae: This family is polyphyletic in Hirayama’s (1998) study: besides the true *Plesiochelys* skull, the coding of cranial characters is possibly based on the unique skull specimen of *Portlandemys* (see Gaffney, 1975a, b, 1976) – not confirmed as belonging to the family - and the coding of postcranial characters is possibly partly based on Eurysternidae.

b) *Xinjiangchelys*: The skull of the type species *X. latimarginalis* is unknown, then the skull characters are taken from the material referred to this species by Peng & Brinkman (1993), including other species as synonymies. However, we are not sure that all this material is truly conspecific or congeneric, according to the different characters in the carapace. It is unclear with which species or specimens are coded the postcranial characters.

c) Sinemydidae: They ought to include *Sinemys* *lens* and *S. gamera* but perhaps also *Ordosemys*, according to Bringman & Peng (1993a, b) and Gaffney et al. (1998). But are *Dracochelys* and *Hangaiemys* included, according to Gaffney & Yeh (1992) and Gaffney et al. (1998)? Also it should be precised which species are coded for the postcranial characters.

d) Chelydridae: the European genus *Chelydropsis* (and its close relative or synonym *Macrocephalochelys*) is not correctly coded. The definition of the family is restricted to the characters used by Gaffney (1975c), whereas the related works of Broin (1977), Mlynarski (1980), and Lapparent de Broin (2000b), with the complete *Chelydropsis* group description, are not cited. Moreover, the inclusion of *Platysternon* in this family is controversial (Joyce, 2007; Lapparent de Broin, 2000b).

e) According to Lynch & Parham (2003) and Parham (2005), specimens attributed to *Osteopygis* *sensu* Hirayama (1998) and Hirayama & Tong (2003) represent a chimera and correspond to two distinct taxa *Osteopygis* *sensu stricto* (postcranial elements belonging to a ‘Macrobaenid’ form) and *Euclastes* (skull). We agree with their suggestion (see Jalil et al., 2009).

f) The taxon Chelomacryptodira Gaffney, 1984, modified in Gaffney & Meylan (1988) and later works, includes extant: a) Trionychoidea (Trionychia: Trionychidae and Carettochelyidae), as well as Kinosternoidea (Dermatemydidae and Kinosternidae) (Joyce, 2007); and b) Testudinoidea (Emydidae and Testudinidae s. l.) (Hirayama, 1985; Bour & Dubois, 1986). It is a large taxon that includes nearly all extant continental Cryptodira, with a long evolutionary route, from Late Jurassic to present. The taxa included in this group are not specified in the H and KL analysis. The monophyly of the group is weakly supported and in contradiction with molecular studies such as the work of Near et al. (2005). The result of the KL analysis is a high number of polymorphisms and of homoplasies with members of the ingroup for many characters. As a whole, the main problem concerns on one hand, the relationships of the Testudinoidea with Chelydridae or with Trionychoidea, and, on the other hand, the relationships of *Platysternon* with Testudinidae and Chelydridae. We consider that *Platysternon*, although being related with Chelydridae, should be removed into a different family: the Platysternidae Gray, 1869 (Joyce, 2007; Lapparent de Broin, 2000b). We have no definitive opinion about the position of the Testudinoidea (including or not the Lindholmemydidae) and the characters uniting them with Trionychoidea as well as with Chelydridae. Anyway, Chelomacryptodira are early diversifed in relation to Chelonioidea and are thus useful for comparison of possible presence of homoplasies.

It is noteworthy that, at the Chelonioidea basal node, the only homoplasy existing between chelomacryptodirans and the ingroup is character 66 (humerus longer than femur - char. 66/1) that characterizes all Chelonioidea; in Chelomacryptodira, a longer femur (or a femur as long as humerus) is attested only in Carettochelyidae, but in the very different context of a flexible limb, contrasting with the rigid paddles of Chelonioidea. Furthermore, as said above, within Chelomacryptodira, the group raising problem is not Carettochelyidae (uncontested sister group of Trionychidae in Trionychoidea), but Testudinoidea. Chelonioidea are well defined by apomorphies of the basicranium not shared by Chelomacryptodira and the other homoplasies are scattered either between Cheloniidae and Chelomacryptodira, or between Dermochelyoidae and Chelomacryptodira, or between the two chelonioid groups, without any influence on the taxa group organization.

**1.2. Problems of the Ingroup**

Several taxa are used at the genus level.

a) For *Euclastes* (separated from *Osteopygis*), it is not specified whether part of the postcranial coding takes into account *Erquelinnesia*, which is different from *Euclastes* in some skull characters (character 6 for example).

b) *Chelonia* (*C*. *mydas*) is coded as representative of the five living genera, after modification of one character that restores the coding of Hirayama (1998) (see characters).

c) It should be noted that the extensive material of marine chelonians studied by Hirayama (1992, 1995), Hirayama & Chitoku (1996), and Hirayama (1998) (in particular for *Santanachelys* and *Notochelone*) has not been described, cited, or figured in detail.

**2. Character definition and polarization**

The characters from Hirayama (1998) are not developed or figured with enough detail and, for some, are hard to understand. Kear & Lee (2006) proposed more comprehensible definition of some Hirayama's characters, and Joyce (2007) provided a clarification of a part of these characters. However, as the matrix of Joyce is not dedicated to marine forms, many taxa from the matrix of Hirayama are not included (only five chelonioids on 64 taxa) and many useful characters are omitted (most chelonioid characters included concern the postcranial). Notably, the only analyzed protostegid is *Santanachelys* that is not representative of the family, taking into account the restricted choice of characters choosen.

Marine forms exist since the Late Jurassic, even if no taxa of this age are included in the matrix. Indeed, *Neusticemys* Fernández & Fuente, 1993 from the Tithonic of Neuquén of Argentina, and new taxa from the Kimmeridgian-Tithonic of Bavaria, Germany (BSPG, München) exist, but until now they were not included in any phylogenitical analysis (Fuente & Fernández, 2011; Lapparent de Broin, 2001). Beside, the separation of Chelonioidea from Chelonomorpha occurs necessarily earlier than the Early Cretaceous because a trionychoid carettochelyid has been reported in the Late Jurassic-Cretaceous boundary of China and Trionychoidea are already much diversified in the earliest Cretaceous (Broin, 1977; Meylan & Gaffney, 1992; Renous et al., 2008; Young & Chow, 1953).

Marine forms are represented in the analysis by two groups revealing separate clades: 1) Early to Late Cretaceous Protostegidae, and fossil and extant Dermochelyidae, with the Cretaceous forms *Bouliachelys* and *Ocepechelon* (= Dermochelyoidae of this work); 2) Cheloniidae. This implies a 30 million years ghost lineage for Cheloniidae, as the oldest preserved forms of Cheloniidae (*Toxochelys*, *Ctenochelys*) are much younger (Santonian) than the first Dermochelyoidae (Albian): is a diphyletism of Chelonioidea possible?

We tried to solve the problems in two ways:

1) We established new polarities when recoding all the characters, according to their evolutionary state, state 0 being always considered as the primitive condition. In particular, a new polarity of characters 1, 4, 90, 92, 93 affects all the taxa where the character is preserved.

2) We used a hypothetical taxon with all characters coded at state 0. Such a taxon is currently used in recent analyses (Brinkman et al. 2009; Joyce, 2007; Parham & Pyenson 2010).

With this method the results indicate clearly where the reversions are and if they are congruent with the current knowledge of the turtle evolutionary history. The changes in polarities are made according to older forms than the outgroups of Hirayama (1998), when they present an opposite polarization, for example the oldest Mesozoic taxon, i.e. *Proganochelys* Baur, 1887 (in Gaffney, 1990). We have also examined polarities using early taxa such as *Kayentachelys* Gaffney et al., 1987 (also Sterli & Joyce, 2007; Gaffney & Jenkyns, 2010; Joyce & Sterli, 2010), new Asian taxa (*Annemys* Sukhanov, 2000; Sukhanov & Narmandakh, 2006; *Heckerochelys* Sukhanov, 2006), and *Eileanchelys* Anquetin et al., 2009 (Anquetin, 2010), as well as some baenids and pleurosternids. Additionally we also compared the included taxa with Pleurodira, the other chelonian suborder (references in Lapparent de Broin, 2000a; Lapparent de Broin et al., 2007; Gaffney et al., 2006).

In some cases, when the character was not applicable in outgroup taxa, we preserved the polarization of Hirayama (1998). Some other modifications concern the polymorphism, which is avoided as much as possible by the retention of the most primitive state of the character (when it is known) in the clearly monophyletic taxa.

**2.1. Outgroups**

a) All the characters of the hypothetical taxon are coded 0.

b) The Plesiochelyidae are restricted here for skull characters to *Plesiochelys solodurensis* (*P.* *etalloni* in Gaffney, 1975a, 1976) and *P*. *planiceps,* (as described in Gaffney, 1976), and for postcranial characters to *P.* *etalloni*, *Craspedochelys* (s. l.), *Tholemys*, and *Tropidemys*, which introduces some modifications compared to Lapparent de Broin et al. (1996) and Lapparent de Broin (2001). For the postcranial material, we followed the coding of Hirayama, although it is possibly, at least partly, based on Eurysternids or non plesiochelyid taxa from Asia.

c) *Xinjiangchelys* and the Sinemydidae (monophyletic?) have been considered as in the KL analysis, except the few polarity changes cited above, applied to all the taxa of the matrix.

d) The Chelydridae are modified with the introduction of *Chelydropsis* (see Broin, 1977; Lapparent de Broin, 2000b), which presents some more primitive states than in coded taxa of the family. We also removed *Platysternon,* the only known member of the Platysternidae, a derived form that introduces additional polymorphism.

e) *Osteopygis* (already separated from *Euclastes* by Kear & Lee, 2006) has been removed from the analysis as it is represented by postcranial elements not referable to Chelonioidea. It has been replaced by *Euclastes*, which is represented by cranial and postcranial material (Hay, 1908; Jalil et al., 2009; Parham, 2005).

f) The Chelomacryptodira include many polymorphisms, due to the complexity of the history of the continental members of this group, which is possibly paraphyletic. The introduction of Mesozoic taxa, currently missing in the analysis, will allow to determine the plesiomorphies of the group (if monophyletic) and to delete at least a part of the polyphyletic characters. As a result, we just changed the few polarities mentioned above (changed for all other taxa). It should also be pointed out that, if the polymorphism concerns almost all characters, the parsimony programs treat the polymorphism as a question mark.

**2.2. Ingroup**

The taxa of the ingroup are the same than in KL and H, with the addition of *Ocepechelon*.

**3. Results**

**Figure S1.1.A -** Matrix with all the KL taxa (re-polarized and adjusted) and the hypothetical outgroup. Strict consensus tree of nine trees (L = 234, Ci = 50, Ri = 69).

The nine trees show variations about the hypothetical relationships of, on the one hand, Chelydridae, Chelomacryptodira, and marine forms, and, on the other hand, *Ocepechelon*, *Bouliachelys*, and *Santanachelys*, with Protostegidae. An interesting point is the irresolution for Chelydridae, Chelomacryptodira, and marine forms, which reflects the problem of the origin of marine forms. Three clades are unvariant: a restricted cheloniid clade (*Puppigerus* + *Chelonia*), the Dermochelyidae, and the Protostegidae without *Santanachelys* (see nodes in figure).

The closer relationships of *Ocepechelon* to Dermochelyoidae than to Cheloniidae is another result of this analysis (discuted below). However, this analysis shows an unlikely system of multiple reversions and homoplasies (as in KL results). Unlikely reversions affect the characters 2, 6, 7, 8, 15, 16, 17, 18, 21, 23, 31, 32, 37 and possible reversions affect characters 3, 4, 5, 12, 28, 41, 42, although some of these characters may constitute strong synapomorphies in other combinations (see below). Character 30 is problematic for its reversion in Protostegidae. Synapomorphies are also unlikely, with many re-acquisitions, except those: a) uniting marine turtles and found in all the analyses with state 1 (see cladogram C and Figure S1.2) that are 14, 29, 33 (except for *Ocepechelon*, see below) and 66; b) 10 and 11 uniting Dermochelyoidae, and 20 uniting Protostegidae with *Ocepechelon* and *Bouliachelys*; c) 35 uniting Dermochelyidae; d) 25 and 26 uniting Protostegidae; e) 96 uniting *Chelosphargis* and *Protostega*; finally, f) 53 and 70 uniting *Puppigerus* and *Chelonia*. We agree with the fact that some of these characters are homoplasies with one or another of the outgroups.

**Figure S1.1.B -** Matrix modified from KL with only the hypothetical taxon as outgroup. Strict consensus of three trees (L=194 Ci=57 RI= 64).

There are four taxa of unresolved marine forms: *Bouliachelys* and *Ocepechelon,* the Protostegidae (including *Santanachelys*), and a surprising supergroup, including the Dermochelyidae and the Cheloniidae.

This tree is not convincing with regard to the other ones obtained (see below), because it is not well rooted: unlikely reversions remain, which affect cranial characters 7, 10, 11, 16 (for *Toxochelys* only), 17, 20, 21 (for *Toxochelys* and *Ctenochelys*), 23, 32, and 34 (for *Toxochelys* only). However, some groups are united by strong skull autapomorphies, beside some seemingly more or less problematic reversions or homoplasies: Protostegidae by characters 9 and 24 and Cheloniidae by characters 23, 32, and 47, with a controversial position of *Ctenochelys* and *Toxochelys*. Like in A, a reversion affects the character 33 in *Ocepechelon* (loss of height of the *dorsum sellae*).

**Figure S1.1.C -** Matrix modified from KL with the hypothetical taxon and Chelomacryptodira as successive outgroups. 4 trees are obtained, two of which are presented here: C1 (strict consensus tree: L = 208, Ci = 54, Ri = 62); and C2 (tree 4 of 4: L = 202, Ci = 55, Ri = 64).

We agree with the principal phylogenetical relationships proposed in these trees, the main result being the Cheloniidae as sister-group of a clade (the epifamily ‘Dermochelyoidae’ named in Bour & Dubois, 1986, corr. Gaffney & Meylan, 1988) including Dermochelyidae, Protostegidae, *Ocepechelon*, and *Bouliachelys*. Protostegidae are well resolved and *Bouliachelys* appears as the basalmost taxon of the ‘Dermochelyoidae’ clade. Changes only concern: a) the relationships of *Toxochelys* with *Ctenochelys* and the other Cheloniidae. We agree with *Toxochelys* as the most basal cheloniid, but its close relationship (or not) with *Ctenochelys* is debatable and outside the scope of this work except for character 14 (see Figure S1.2 below); and b) the problematic position of *Ocepechelon* within the Dermochelyidae, a poorly resolved clade because of unsufficient material.

Most of the reversions seen in the first analysis are absent; only four problematic reversions remain, concerning both postcranial elements (52/0 and 54/0) at the base of Dermochelyoidae (except *Bouliachelys*) and the skull (15/0 and 30/0):

1) Character 15/0: The palatine is not included in the triturating surface, independently for *Ocepechelon* and Protostegidae (except *Santanachelys*). This reversion is difficult to explain in each case, as well as its loss if both taxa had an ancestor with a secondary palate involving the palatine in its triturating surface. The inclusion of the palatine into the triturating surface has been acquired several times in both the outgroups and the marine forms, independently of the occurrence of a secondary palate (see character list). There is thus a confusion between the condition ‘palatine participating to the posterior part of the triturating surface without secondary palate’ and the condition ‘palatine participating along to a triturating surface integrated in a secondary palate’. In our opinion, a new homoplasic acquisition is more likely than several losses.

2) Character 30/0: In Protostegidae, the *foramen posterius canalis carotici interni* is located anteriorly, visible in ventral view at the basisphenoid-pterygoid limit (and not posteriorly in the pterygoid). Even the extant *Dermochelys*, which is a fewly ossified paedomorphic form, has a carotid entering the skull posteriorly in the pterygoid. In this case, the particularity lies in the absence of ossification of the carotid canal roof, resulting into the fact that the carotid canal and the *canalis cavernosus* are separated by soft or cartilagineous tissue, not by bone. It is therefore not an absence of carotid canal as interpreted by Kear & Lee (2006). The reversion to a very primitive state for turtles as soon as the Albian *Santanachelys* in Protostegidae, which is the oldest and most primitive one, is difficult to understand.

Another problem is the loose relationship of *Ocepechelon* with Dermochelyidae, based on 3 characters:

1) Character 1/1: The highly homoplasic loss of dermal scutes, which is shared here by *Ocepechelon*, *Dermochelys*, and *Protostega*, as well as some Cheloniidae, is possibly not systematically a true loss because scutes are sometimes so thin that their limits are not marked on bone (see the character list). Additionally, it is shared by some Chelomacryptodira, including the Trionychia (true loss in soft-shelled turtles Trionychidae and Carettochelyinae, reduction in Anosteirinae).

2) Character 21/1: The complete absence of the *foramen palatinum posterius* is shared here by *Ocepechelon*, *Dermochelys*, *Protostega*, Cheloniidae (except the basal *Toxochelys*, *Ctenochelys* and *Allopeuron* (only a lateral fissure), and the outgroups (Plesiochelyidae and some Chelomacryptodira), although this disappearance is not truly homologous in all cases (see List of characters)*.*

3) Character 27/1: The character '*processus trochlearis oticus* with small participation from prootic to the trochlea prominence', present in Dermochelyidae, appears as insufficiently defined for a good application to *Ocepechelon* in relationship with Dermochelyidae. It is also possibly not well coded in *Corsochelys* and *Mesodermochelys*.

Indeed, the prootic of *Corsochelys* (in Zangerl, 1960) is prominent with a concave dorsal surface, contributing to the trochlea. However, as the quadrate is not preserved, its relative participation to the trochlea remains unknown. The prootic of *Mesodermochelys* (in Hirayama & Chitoku, 1996) is an isolated bone. It seems also to exhibit a protuberance with a concave dorsal surface, contributing to the trochlea. Therefore, both *Corsochelys* and *Mesodermochelys* seem to show a prootic participation to the trochlea and to its protuberance, contrarily to *Ocepechelon*.

As far as they are concerned, *Dermochelys* (weakly ossified, without trochlear protuberance), and *Ocepechelon* (strongly ossified at the trochlea level) exhibit so different cranial constructions that it appears impossible to define a homologous (prominent or not) ‘prootic participation to the trochlea’.

*Dermochelys* possesses an unossified anterior area of the cerebral cavity that remains cartilagineous. As a result: 1) the bony *processus inferior parietalis* and epipterygoid are completely reduced; 2) there is no bony wall to surround the trigeminal nerve V foramen, nor to delimit the carotidian canal and the *canalis cavernosus* (resulting in a completely opened area including these three structures); 3) the *rostrum basisphenoidale* and the *trabeculae* are not ossified but present as cartilage; 4) the prootic and quadrate are juxtaposed in an inclined linear wall, roofing the cranio-quadrate passage (including the carotid canal inferiorly) medially and the inner ear laterally. As in *Proganochelys*, no bony protuberance indicates the location of the trochlea in relation to the prootic and quadrate, although a *cartilago transiliens* has been observed in the living animal. To sum up, it is impossible to estimate the prootic participation to the trochlea in *Dermochelys*.

*Ocepechelon* has a normal ossification of the anterior cerebral cavity. The prootic is partly raised vertically below the *processus inferior parietalis* and it shows roughness at its base, at its boundary with the quadrate, at the beginning of a very wide and strong trochlea. The quadrate trochlea is well ossified and very rough, transversal and not inclined (in relationships with the flattened and widened skull shape), wide from the lateral wall of the skull up to the quadrate-pterygoid suture, with well marked angles; its dorsal face is concave (not highly dorsally protuberant), but its protuberance is more visible in ventral view (with an inner protuberant angle slightly broken): there is no participation of the prootic to the trochlea, but in a different way than in *Dermochelys*. Finally, the ‘trochlear dorsal concavity’ is located on the prootic in *Corsochelys* and *Mesodermochelys*, and on the quadrate in *Ocepechelon*.

To sum up, the identical coding of this character in Dermochelyidae and *Ocepechelon* is incorrect as the character is not well defined and probably not homologous in all concerned taxa. The cryptodiran trochlea shows a great diversity of shapes in height (degree of protuberance), in width, in convexity, linearity or concavity, in the location of the protuberance (medial, lateral or both), in the occurrence or not (when this area remains mostly or completely cartilaginous) of marked roughness, and finally in the relative participation of the prootic and quadrate to the trochlea and its possible prominence (for examples, see Gaffney, 1979; Lapparent de Broin, 2004). Character state 27/1 is therefore obviously not a strong synapomorphy shared by *Ocepechelon*, *Corsochelys,* *Mesodermochelys*, and *Dermochelys*, and it should be better defined in future analyses.

The phylogenetic position of *Corsochelys* could be reconsidered. Zangerl (1960) presented this genus as an advanced cheloniid, but its anatomy offers various possibilities. The humerus great trochanter is barely developed, as in toxochelyids (sensu Zangerl, 1953b), and the radial smaller trochanter, although damaged, may not present the dermochelyid apomorphy; the anterior border of the carapace is much more protruded, as in modern cheloniids, than in the protostegid *Desmatochelys* (Zangerl & Sloan, 1960); the morphology of other shell elements is not conclusive (for example the hyoplastron, which is indented like in some cheloniids); finally the scapula-acromion angle is relatively open (ca. 115°), but not the highest. *Corsochelys* is related with *Dermochelys* and *Mesodermochelys* by skull elements that are incomplete, and in particular the basiphenoid area and the prootic. The main shared character is the assumed reduction of the trabeculae (35/1). Indeed, the dorsum sellae is high with a sagittal crest on a prominence between its upper posterior border and the sella turcica, as in advanced Cheloniids and *Dermochelys*, but in front, the trabeculae end anteriorly thinned and are not abruptly interrupted like in *Dermochelys* by the lack of ossification (with a cartilagineous prolongation). It is therefore difficult to detect if they are damaged (the most probable situation) rather than weakly ossified in *Corsochelys*. Anyway, the configuration is distinct from that of *Ocepechelon*.

**Figure S1.2 -** The relationships of *Bouliachelys* and *Ocepechelon* with Dermochelyidae and Protostegidae within the clade ‘Dermochelyoidae’ are not resolved. Only the synapomorphies considered as accurate (black circles), and apomorphies with admitted homoplasies and admitted reversions (open circles) are presented here, taking also into account the homoplasies with the other outgroups (Figure S1.1, A and B).

Chelonioidea, which include Cheloniidae as sister-group of the large clade ‘Dermochelyoidae’ (with *Bouliachelys*, *Ocepechelon*, Dermochelyidae, and Protostegidae in an unresolved polytomy), share the following synapomorphies: **14/1**, *foramen praepalatinum* absent. The character has been coded as absent (1) in *Toxochelys* by Hirayama (1998), followed by Kear & Lee. The foramina are not clearly visible on the photographs of the crushed *Toxochelys latiremis* skulls (Zangerl, 1953b, pl.12 and pl.13) but they are present in the reconstructed figure of Zangerl (1953b, fig. 60), so that it will be necessary to verify the character in the original *Toxochelys* material which includes several species*.* The change in the matrix does not modify the results in the trees such as that presented in Fig. S2.1, C2. In the consensus (Fig. S2.1, C1), *Toxochelys* stays in irresolution with *Ctenochelys* and the group of following cheloniids. The character appears as reversive in *Toxochelys* and could be deleted from the characters defining the Chelonioidea in Figure S1.2; it may be estimated as a homoplasy between further cheloniids and Dermochelyoidae. **29/1**, both *foramina anteriora canalium carotici interni* close together in the *sella turcica*. **33/1**, high *dorsum sellae*. A reversion is observed with this matrix in *Ocepechelon*, which has a flattened skull that induces both flattening and widening of the cerebral cavity. **66/1**, humerus longer than femur. This character is homoplasic in Chelomacryptodira (*Carettochelys*) (a non-marine but highly swimming form with flexible paddles derived from those of the Trionychid type, instead of having rigid paddles like in open-sea living chelonioids); according to KL matrix, it is also present in some Sinemydidae (0,1), which are also non-marine taxa without rigid paddles, but the derived character state presence is not confirmed. It might also have appeared several times in marine chelonioids, linked with the homoplasic paddle development. This character is not preserved in *Ocepechelon*, but probable, taking into account the large number of skull characters related to a marine life. It should be noted that large cryptodiran girdles, humeri, femora, and carapace elements, from coeval late Maastrichtian phosphatic levels of Grand Daoui, are preserved in the OCP collection (Khouribga, Morocco); they could partly correspond to *Ocepechelon* by their large size and morphology (see ‘Detailed description’) as well as to another large chelonioid.

The clade Cheloniidae is better analysed with more taxa in more recent works than Kear & Lee, 2006 (references in Parham & Pyenson, 2010) but the results are identical as a whole. Here: Cheloniidae (*Toxochelys* (*Ctenochelys* (*Euclastes* (*Puppigerus*, *Chelonia*)))) are diagnosed by the following characters: **2/1**, nasal absent. This character is homoplasic in *Dermochelys* (although the original location and shape of the nasal are still clearly visible on extant skulls), *Protostega*, and Chelomacryptodira. **18/1**, vomer-palatine contact anterior to internal nares (choanae) present (secondary palate first step). In *Toxochelys*, the character could be weak to nearly absent (Zangerl, 1953b, p. 174). In *Dermochelys*, this contact is often incomplete or inequal from one side to another. **31/1**, ventral surface of basisphenoid with a V-shaped crest. This character is homoplasic in *Bouliachelys*. **47/1**, ventral keel on posterior cervical centra present. This character is homoplasic in some Chelomacryptodira (0,1). **54/1**, anterior articulation of first thoracic vertebra facing ventrally or anteroventrally. This character is homoplasic in Chelomacryptodira. **63/1**, thyroid fenestra large and unique. This fenestra is large, but still divided by a complete thin blade of bone in *Allopleuron* while in living cheloniids, the thyroid fenestra is divided by a long cartilage; whereas in *Dermochelys* and *Mesodermochelys* there are two small fenestrae, separated by bone, as in protostegids. This character is homoplasic in various Chelomacryptodira (0,1). **74/1**, ulna-radius contact present through rugosity and ridge. This character is homoplasic in Chelomacryptodira terrestrial Testudinidae (a better definition of this character is needed to evidence the pecularities of each taxon). **85/1**, nine neurals. As a matter of fact, the number is variable in Cheloniidae not included in the analysis (Parham & Pyenson, 2010). This character is homoplasic in *Protostega* and not applicable for the unossified shell of *Dermochelys*.

The clade (*Ctenochelys* (*Euclastes* (*Puppigerus*, *Chelonia*))) shares two synapomorphies and one homoplasy: **23/1**, pterygoids with a median ventral ridge. Although partly medially attenuated in *Chelonia* it is often still present and sometimes the crest is also reduced in *Caretta*. As a matter of fact, it characterizes only a part of cheloniids beside the modern taxa (Parham & Pyenson, 2010). **32/1**, basipterygoid processes of basisphenoid with posterolateral projections and V-shaped basisphenoid. **34/1**, trabeculae close together (even, more or less flat rods) or fused (rounded rod), as in *Bouliachelys*, *Ocepechelon*, and Protostegidae (not applicable in unossified *Dermochely*s, poorly known in *Corsochelys*, and unknown in *Mesodermochelys*). This character is insufficiently known for an accurate definition.

The clade (*Euclastes* (*Puppigerus*, *Chelonia*)) is only defined by homoplasic characters: **17/1**, vomers developed into a narrow sagittal pillar (essential for a secondary palate). Not identical, a vomer pillar exists in *Dermochelys*, but is very short because of the raised and vertical ventral vomer face. Its presence in *Protostega* is not impossible, as this taxon is figured with a kind of short secondary palate in Hirayama (1995) but without any clear indication of the palatines expansion. **21/2**, *Foramen palatinum posterius* absent. This character is homoplasic in *Ocepechelon*, *Dermochelys*, and *Protostega,* but in Cheloniidae this foramen disappears during the secondary palate development; however in Dermochelyoidae it is progressively reduced by a lateral opening, while in Cheloniidae the jugal remains ventrally linked to the pterygoid whereas the foramen reduces and the two characters have to be associated. The character is also homoplasic in some Chelomacryptodira.

The clade (*Puppigerus*, *Chelonia*) is characterized by characters that remain unknown in *Bouliachelys* and *Ocepechelon*: one true synapomorphy 7**0/1** (lateral process of humerus V-shaped) and two homoplasic synapomorphies: **53/1** (double articulation between the 7th and 8th cervicals present), which occurs also in some Chelomacryptodira. **61/1** (angle between main shaft and acromion of the scapula > 100-110°) is present in the clade (*Puppigerus, Chelonia*), and it is homoplasic in Dermochelyidae and Protostegidae. *Bouliachelys* and *Ocepechelon*, in which this postcranial character is unknown, probably shared this character, like all fully marine large turtles possessing a paddle. Concerning *Ocepechelon*, it should be noted that a larger angle (120-130°) is observable on large scapulae associated to large humeri from the Late Maastrichtian of Grand Daoui (Oulad Abdoun Basin, Morocco) (see above and ‘Detailed description’). The character may be sometimes difficult to apply (see List of characters) and its definition has been modified in other cheloniid analysis (Parham & Pyenson, 2010).

The clade ‘Dermochelyoidae’ (*Bouliachelys*, *Ocepechelon*, Dermochelyidae (Protostegidae)) is characterized by the following characters: **10/1**, medial process of jugal beneath orbit weakly developed or absent. **11/1**, jugal-pterygoid contact absent. Both characters represent steps for the reduction and disappearance of the *processus pterygoideus externus* and for the following character. **21/1**, *Foramen palatinum posterius* opened posterolaterally, but anteriorly forming an embayment in the palate. It appears here as a first step for the complete disappearance of the *foramen palatinum posterius* by lateral regression of the lateral mid-palatal surface, and not by its inclusion in a large secondary palate as in cheloniids, with preservation of the jugal-pterygoid contact.The position of the state 21/1 at this node, from which state 21/2 is derived independently in *Dermochelys* and *Protostega*, is an optimization. The character should not be situated at this unresolved node, being unknown in *Corsochelys* and *Mesodermochelys*, so that state 2 could be directly developed from state 0 in *Dermochelys* and developed from state 1 (acquired by protostegids) in *Protostega*. The unresolved position of *Bouliachelys* (state 1) and *Ocepechelon* (state 2), both lacking postcranial material in the analysis, is responsible for the optimization of the cladogram. Like characters 10 and 11, it is linked to the disappearance of the *processus pterygoideus externus* and of the *foramen palatinum posterius* (without secondary palate integrating the foramen, contrary to Cheloniidae). **20/1**, palatines meeting medially, vomer not contacting pterygoid. This character, unknown in *Corsochelys* and *Mesodermochelys*, shows a reversal in *Dermochelys*. We agree on that, because in *Dermochelys* the pterygoids are anteriorly much protruding between the palatines to contact the vomer, instead of a prolongation of the vomer up to the pterygoid. It appears as derived by comparison with all other cryptodira (including the Mesozoic forms) sharing the contact between the two bones. Although this anterior protrusion of the pterygoid up to the vomer is roughly identical to that in *Proganochelys*, it is achieved in this taxon in a more primitive palatal context, with the interpterygoid vacuity in which the parasphenoid rostrum is anteriorly extended. The characters **10**, **11**, **20**, and **21** are also homoplasic in some Chelomacryptodira (as in Plesiochelydiae – see matrix with complete outgroup set), reflecting a palatal adaptation in connection with the feeding technique. As they are not present in Cheloniidae, they are the best characters for the Dermochelyoidae definition. Characters **34/1** and **61/1** have been commented above; they are homoplasic in Cheloniidae.

The clade Dermochelyidae suffers from the lack of many skull characters in *Corsochelys* and *Mesodermochelys*, a problem that might be partly resolved introducing Cenozoic taxa, but the lack of postcranial material remains problematic for *Bouliachelys* and *Ocepechelon*. The problematic position of *Corsochelys* has been evoked above (Figure S1.1, C). The only synapomorphy shared by *Corsochelys* and *Dermochelys* is **35/1** (reduced *rostrum basiphenoidale*), which is highly debatable, the rostrum being eroded in *Corsochelys*. It also occurs in Sinemydidae (see matrix with complete outgroup set), but we cannot tell if it is a paedomorphic complete absence of ossification, as in *Dermochelys*, or, more probably, the primitive presence of a short rostrum. Character **34/0** (wide separated trabeculae) appears as reversive; it may reflect either the regressive ossification in *Dermochelys*, or a primitive condition in *Corsochelys*. The absence of scutes (**1/1**), in correlation with characters **80/1** and **91/1** (absence of carapace and plastral scutes), is either widely homoplasic, or not well stated for every turtle in which the scutes are thin and do not let prints in the bones (especially possible in *Corsochelys*). It is one of the characters ambiguous for the position of *Ocepechelon*, as a sister taxon of dermochelyids or as a sister taxon of *Protostega*. However, the characters **80/1** and **91/1**, concerning postcranial elements, are unknown in *Ocepechelon* and *Bouliachelys* and are homoplasic in various Chelomacryptodira. The small contribution of the prootic to the trochlea (**27/1**) has been discussed above. **43/1** (dentary posteriorly expanded, almost reaching the articular surface) is unknown in *Corsochelys* (and *Ocepechelon*). This character is homoplasic in some Chelomacryptodira. **73/1** (prominent anterior projection of lateral, i.e. radial, process of humerus) characterizes *Mesodermochelys* and *Dermochelys*: unknown in *Ocepechelon* and *Bouliachelys*, it is neither present in *Corsochelys* nor in the large humeri of the Late Maastrichtian from Grand Daoui, (see ‘Detailed description’).

Apomorphies of *Dermochelys* have been shown above as homoplasies (2, 17, 18, 21). **22/1** (*processus pterygoideus externus* without vertical flange) accompanies the complete loss of the *processus pterygoideus externus* and, once more, reveals the ambiguous position of *Ocepechelon*, being also present in *Dermochelys* and *Protostega*. The reversion **20/0** (palatines not meeting medially) has already been admitted for *Dermochelys*, in which it constitutes an autapomorphy.

The clade Protostegidae is defined by clear synapomorphies: **9/1**, jugal-quadrate contact present. **24/1**, pterygoid taking part into the mandibular condyle. **71/1**, expansion of the lateral process of the humerus onto the ventral surface (a not well defined character). **75/1**, radius curves towards anteriorly.

The clade (*Notochelone* (*Desmatochelys* (*Chelosphargis*, *Protostega*))) exhibits the following synapomorphies: **72/1**, medial concavity of the lateral process of the humerus present. **104/1**, short xiphiplastra with lateral curvature present. There are some homoplasies: **19/1**, lingual ridge of the maxilla strongly developed (homoplasic in Plesiochelyidae, see matrix with complete outgroup set, and in *Bouliachelys*). **91/1**, plastral scute sulci absent or rudimentary (see above and List of characters, characters 1, 80, and 91).

The clade (*Desmatochelys* (*Chelosphargis*, *Protostega*)) is defined by several characters: **25/1,** pterygoids form an extremely narrow bar. Extremely narrow in *Protostega*, the pterygoids are progressively wider in other taxa and the limit with ‘at least moderately wide’ is difficult to delimit (see Hirayama, 1995, and List of characters). **26/1**, *processus trochlearis oticus* with small contribution from quadrate.

*Chelosphargis* and *Protostega* share the character **96/1** (plastron star-shaped). This character should be more precisely defined to be more accurately compared with the hyoplastra and especially the strongly indented and long hypoplastra found in the Late Maastrichtian Phosphates of Morocco. The hyo-hypoplastra are not deeply indented in protostegids, forming a massive quadratic plastral median part with short oblique to short very oblique xiphiplastra (Hirayama, 1995; Hay, 1908; Wieland, 1909 (**104/1**). The hypoplastra (and xiphiplastra) are more elongated in others chelonioids with more indented elements, such as those of Grand Daoui (see ‘Detailed description’) and various cheloniids.

*Protostega* shows a suite of homoplasic characters already discussed (**1/1**, **2/1**, **21/2**, **22/1**, **80/1** and **85/1**). Some of those are shared with *Archelon*. Other possible synapomorphies have to be searched for with a new observation of the material.

From this analysis it is clear that the positions of *Bouliachelys* and *Ocepechelon* attract attention, occupying a basal unresolved position within the ‘Dermochelyoidae’, with which they share the apomorphies **10/1**, **11/1**, and **20/1**.

*Bouliachelys* does not share any unambiguous character with Dermochelyidae. With Protostegidae closer to *Protostega* than to *Santanachelys*, it shares the character **19/1 (**lingual ridge of maxilla strongly developed), and exhibits one Cheloniidae character: **31/1 (**ventral V-shaped crest on the basisphenoid surface). The absence of postcranial characters may explain the irresolution.

The phylogenetic position of *Ocepechelon* is ambiguous due to a mixture of characters of both Dermochelyidae and Protostegidae. The absence of postcranial characters is also implicated in its position. With *Dermochelys* and *Protostega*, it has been shown that the loss of scutes (**1/1**) and the complete reduction of the *foramen palatinum posterius* (**21/2**) are common characters. Also, the absence of participation of the prootic to the trochlea (**34/0**, **35/1**) is not homologous with that of *Dermochelys*, and *Ocepechelon* does not present any evidence of ossification reduction. *Ocepechelon* shares some characters with *Protostega*, but not the unambiguous synapomorphies of Protostegidae, such as 9/1 and 24/1. Obviously, some characters are not available in *Ocepechelon* (skull: 36, 37, 38; lower jaw and postcranial elements). On marine cryptodirans, key-characters concern particularly the appendages (girdle and forelimb); this explains the impossibility to fully resolve the problem of the relationships *Ocepechelon*, as long as its postcranial elements are not identified. For the carapace, it should be noted that the absence of scutes is highly probable. The main shaft / acromion angle of the scapula is expected to be more than 90°, around 120-130°. The humerus is expected to exhibit at least some character states of marine chelonioids closer to *Chelonia* than to Toxochelyidae (sensu Zangerl, 1971), which are: flattened, with a trochanter major much more elongated anteriorly to the head, and a wide and flat distal extremity. It should be pointed out that such large humeri and scapulae have been found in coeval levels of the Oulad Abdoun Basin of Morocco (see above and ‘Detailed description’), associated with shells with moderate lateral fontanelles, an indented nuchal, and highly indented (but neither protostegid nor *Dermochelys* related) plastral elements. All these postcranial elements evidence the occurrence in the Oulad Adboun Basin of large marine chelonioids different from Maastrichtian cheloniids such as *Allopleuron* and *Protosphargis*. *Protosphargis veronensis* has been considered as a potential dermochelyid (Tong & Hirayama, 2004); but the observation of its pelvis (Capellini, 1984, pl. 6) demonstrates its cheloniid affinities as it exhibits a large *fenestra thyroidea*, in contradiction with the two small and well separated fenestrae of protostegids and *Dermochelys*. *Protosphargis*, whose skull remains unknown, has a very indented hyoplastron shortly in contact with the hypoplastron, a character that justified the attribution of Tong & Hirayama (2004). However, the hyo-hypoplastron contact is barely thinner than in *Allopleuron,* in a closer indented morphological context than in *Dermochelys*, which is devoid of indentations*. Allopleuron*, an ‘aberrant cheloniid’ according to Tong & Hirayama (2004), was also considered as a possible dermochelyid before the full description and definitive cheloniid attribution (Mulder, 2003). The Moroccan plastra from Grand Daoui, as well as *Allopleuron* and *Protosphargis*, do not share the wide contact space of hyo-hypoplastral short indentations seen in Protostegidae.

It is clear that *Ocepechelon* represents a new feeding mode that clearly separates it from other chelonioids, but this does not give any information concerning its phylogenetic affinities. *Dermochelys* is the only living form of the Dermochelyoidae clade. If *Ocepechelon* belongs to this clade, it is however not a close relative to *Dermochelys*, their possible common ancestor remaining not defined and surely very ancient. The results of our analysis suggest a provisional monophyly of the marine forms, probably radiating early during the Jurassic, but we failed to settle its origin and relationships with any outgroup of the analysis.

The morphology of marine cryptodirans induces homoplasies around two main axes:

1) Secondary palate: a secondary palate has been acquired several times in turtle history, in pleurodires and in both primitive (such as *Solnhofia*) and derived cryptodires (trionychoids and chelonioids), in sandownids (Tong & Melan, 2013), and in freshwater, littoral, or truly marine forms. For example, it may be independently acquired in Cheloniidae s.l. such as in the *Euclastes* group (Brinkman et al., 2009; Jalil et al. 2009; Parham & Pyenson, 2010), as well as in more advanced Cheloniidae s.s. (insufficiently defined by characters 14 to 18).

2) Paddle: several morphotypes of paddles, from short to very long and rigid ones (flippers), have also been acquired in very diverse turtle groups. The paddle morphology of *Ocepechelon* remains unknown (characters 60, 61, 66 to 78). Although a long paddle is expected in *Ocepechelon*, its highly peculiar flattened skull, with eyes and nares probably laying just above or at the level of the water surface suggesting a very peculiar feeding technique, does not necessarily imply a limb as derived as in extant *Dermochelys*. Due to a very regressive ossification and a particular physiology, this last taxon is able to cross oceans and to follow its prey in streams reaching deep areas of the water column up to 1000 m.

Up to now, *Ocepechelon* constitutes an endemic Moroccan marine chelonioid restricted to the Late Maastrichtian, but it could be related to other contemporaneous African taxa, such as indeterminate taxa based on large humeri from the Benguerir Phosphates (Ganntour Basin) of Morocco and from the Dakhla Oasis of Egypt (Lapparent de Broin & Werner, 1998; Lapparent de Broin, 2000a).

**4. Character list**

We strictly followed the character matrix of Kear & Lee (2006) (KL), so that our character numbers are the same than those of these authors. As Kear & Lee, we also mention the corresponding characters of Hirayama (1998) (H). Unchanged characters from KL are preceded by an asterisk.

**1. Cranial scute sulci on dermal roofing elements. 0: present; 1: absent (H1).** Modification from H and KL (reversed polarity): *Proganochelys* possesses cranial scutes. *Kayentachelys* is too damaged to check the presence or not of a scalation. For *Anemys*, no scalation is mentioned by Sukhanov (2000) and Sukhanov & Narmandakh (2006), and no photographs are provided. Scutes are present in Meiolaniidae (Gaffney, 1983, 1996; Broin & Fuente, 1993), Pleurosternidae, Baenidae, etc. The character is important at the level of marine forms related to *Ocepechelon*, and its comparison with *Dermochelys*, in which no scutes are present, only skin, as presumably in protostegids. The question is to know whether there are really no dorsal roof scutes on *Toxochelys*, *Ctenochelys*, and some other cryptodires. Indeed, sometimes in prepared skeletons of extant taxa and fossils (i.e. Chelydridae and Cheloniidae), scutes are very thin and poorly visible, or they are thick but do not let marked sulci on bones. The same problem of presence/absence of scute sulci exists for the dorsal shell and plastron (characters 80 and 91 respectively).

***2. Nasal. 0: present; 1: absent (H2).** KL mentioned that: ‘*Toxochelys* is coded as lacking nasals, following Hooks (1998) (Appendix 2; H2). This is clearly visible on the holotype (subadult) skull, and on the inner surface of the referred specimen.’ The reduction of the nasals is a common trend in all turtles and we agree with its loss three times in marine forms (as in some Sinemydidae, Chelomacryptodira, and Chelydridae).

***3. Prefrontals. 0: not meeting medially; 1: meeting medially** **(H3).**

**4. Prefrontal-Postorbital contact. 0: present; 1: absent (H4).** Modification from KL (reversed polarity). Contact present in *Proganochelys*. The absence of contact is established as soon as the Early Jurassic and is variable within species. Most extant cheloniids exhibit a frontal reaching the orbit margin except *Caretta* (in most specimens the frontal is excluded from the orbit margin). Contact is present in *Dermochelys*. This character is considered as adaptative, possibly reversible when linked to the widening of the interorbital space for the lateral position of the orbits, diagnostic but without obligatory familial representativity at the phylogenetic level.

***5. Orbit orientation. 0: faces laterally; 1: faces dorsolaterally (H5).** Reversion is possible (linked to the previous character), in particular in *Puppigerus* and *Chelonia* according to the analysis.

***6. *Processus inferior parietalis*. 0: narrow anteroposteriorly; 1: wide anteroposteriorly (H6).** This character is not well suited for *Ocepechelon*: as the cerebral cavity is naturally flattened, an exteriorization of both the dorsal epipterygoid border and the *processus inferior parietalis* is produced that modifies the configuration of the area. State 1 matches the condition of, for example, *Chelydra* (long process extended forward), and state 0 matches the condition of extant cheloniids and *Erquelinnesia* (narrow and located backwards). Note that the *Euclastes* coding does not represent the whole *Euclastes* group as defined by Jalil et al. (2009, including *Erquelinnesia*). In *Erquelinnesia*, the processus is short in front of the Trigeminal nerve (V) foramen, but in *Euclastes acutirostris* it is long, as in *E. wielandi*. The character is not well known in the turtles included in the analysis and should be redefined.

***7. Parietal-Squamosal contact. 0: present; 1: absent (H7).**

***8. Posterior temporal emargination. 0: weak, foramen stapedio-temporale concealed in dorsal view; 1: moderate, f.s.t. but not entire *processus trochlearis* exposed in dorsal view; 2: strong, entire *processus trochlearis oticus* exposed in dorsal view (H8).** We agree, although this character is debatable, because it partially overlaps with character 7.

***9. Jugal-Quadrate contact. 0: absent; 1: present (H9).**

***10. Medial process of Jugal beneath orbit. 0: strongly developed; 1: weakly developed or absent (H10).**

***11. Jugal-Pterygoid contact. 0: present; 1: absent (H11).**

***12. Ventral cheek emargination. 0: absent or indistinct (a shallow concavity); 1: deep or at least well distinct (H12).** KL simplified this character. It is not well defined, without clear separations between states. Not very significant.

***13. Premaxilla. 0: not hooked; 1: hooked (H13).** Note that in *Ocepechelon* there is no hook due to the highly autapomorphic tube-shaped snout, so that this character is coded 0.

***14. *Foramen praepalatinum*. 0: present; 1: absent (H14).** Its presence has to be checked in *Toxochelys.*

**15. Upper triturating surface. 0: not involving palatine; 1: involving palatine (H15).** This character has been recoded for several taxa. Hirayama introduced this character as one of the parameters defining the secondary palate in chelonioids. As it is presented, no discriminating differences have been made between: a) continental forms, which very frequently exhibit (i.e. Chelydridae and various Chelomacryptodira) a participation of the palatines to the triturating surfaces in their mid-posterior part, or a palatine participation to a secondary palate not strictly homologous to that of cheloniids; b) marine forms, which also exhibit a participation of the palatines to the triturating surfaces but only to a secondary palate. The palatine participation to the posterior part of the triturating surface is frequent in turtles and variable in proportion. However, the secondary palate in chelonioids is better achieved only when the palatine is extended anteriorly up to the anterior border of the choanae and contacts the vomer anteriorly (character 18), with the vomer dropped at the secondary palate level below the primary palate by a pillar (character 17). In *Toxochelys*, the palatine and vomer are united but the vomer is not lowered with a longitudinal pillar, and there are no rugosities indicating their participation to the triturating surfaces. In *Ctenochelys*, the secondary palate shows a lowered vomer with rugosities and a very short longitudinal pillar. *Protostega* and *Archelon* are not known enough to be correctly interpreted.

**16. Upper triturating surface. 0: without contribution from vomer; 1: with contribution from vomer (H16).** We have reestablished the meaning of this character (vomer not included / vomer included), to distinguish the continental forms (without a secondary palate) from *Dermochelys* and cheloniids. *Dermochelys* has a very thin and particular secondary palate, with triturating surfaces covered by flexible fleshy cones retaining the prey, linked to its diet constituted mostly of jellyfish (but also other preys and algae). Several skulls of *Dermochelys* (MNHN coll.) show a participation of the palatines to the triturating surface, extended anteriorly up to the anterior border of the choanae, contacting more or less closely the vomer (deficient ossification), as well as a verticalization of the vomer in a short and dorsally inclined sagital pillar. We did not introduce the difference with marine forms, but we separate it from the primitive state in continental forms. To differentiate *Dermochelys* and the other marine chelonioids, it may be necessary to introduce a new character, with the possibility of a homoplasy.

**17. Vomers. 0: not developed into narrow sagittal pillar; 1: developed into narrow sagittal pillar (H17).** Recoded for *Dermochelys*.

**18. Vomer-Palatine contact anterior to internal nare (*apertura narium interna*). 0: absent; 1: present (H18).** Recoded for *Dermochelys*. Coded 0 for *Protostega* by H and KL, which is dubious according to the figure in Hirayama (1995), where a secondary palate is figured, short in agreement with the relatively dorsal position of the external nare, and the palatine suture not completed.

***19. Lingual ridge of maxilla. 0: absent or weakly developed**; **1: strongly developed (H19).**

***20. Palatines and Vomer-Pterygoid contact. 0: palatines not meeting medially, vomer contacting pterygoid; 1: palatines meeting medially, vomer not contacting pterygoid (H20).** Accepted reversion in *Dermochelys.*

***21. *Foramen palatinum posterius*. 0: fully enclosed within palatal bones; 1: open posterolaterally, but anteriorly forms embayment in palate; 2. Absent (H21).** We agree with the KL treatment of this character: ‘States 0 and 2 in H21 were not phylogenetically informative and are combined here as state 0. *Desmatochelys* has a slight embayment (e.g. Hirayama, 1995, 1997) and is coded with state 1.’ However, it should be noted that state 2 of ‘Dermochelyoidae’ is not homologous to that of cheloniids: in ‘Dermochelyoidae’ the foramen disappears by non-ossification of the lateral wall of the anterior palate, due to the jugal-pterygoid contact full reduction (characters 10 and 11), whereas in cheloniids the foramen becomes reduced and disappears with the development of the secondary palate but the jugal-pterygoid contact remains.

***22. *Processus pterygoideus externus*. 0: with vertical flange or small projection; 1: totally lacking vertical flange (H22).** The definition of the state 1 implies a reduction.

This character is not applicable to *Proganochelys*, in which the flange is primitively absent.

**23. Pterygoids. 0: without median ventral ridge; 1: with median ventral ridge (H23)**. KL state: ‘Not present in *Chelonia mydas*, contra Hirayama (1998)’. We disagree with this coding and have reintroduced the state 1 for *Chelonia mydas* (which is representative of the five modern cheloniid genera) because the sagittal ridge is often present though weakened and the crest can be nearly absent in *Caretta* as well.

***24. Pterygoid. 0: Pterygoid. 0: does not form part of mandibular condyle. 1: forms part of mandibular condyle (H24).** Hirayama’s (1998) definition is “0: not reaching the mandibular articular facet of the quadrate; 1: reaching the mandibular articular facet of quadrate” (H24). Actually, it is a pterygoid extension on the quadrate articular process up to the condyle but without participation to the condyle facet (as visible in taxa coded 1). It extends nearly up to the condyle in *Ocepechelon* (coded 0) in an intermediate position, not as extended as in advanced protostegids but that might possibly be conform to that of *Santanachelys* (coded 1 by Hirayama, 1998, but not clearly figured).

***25. Pterygoids. 0: at least moderately wide; 1: extremely narrow bar (H25**). KL state: ‘Hirayama described state 1 as “narrow and C-shaped”, but ‘C-shaped’ pterygoids also occur in taxa coded with state 0 (but with a more or less well shaped C, pers. obs.); this part of the character is omitted here’. We agree with KL coding but it is difficult to delimit states 0 and 1 in some taxa, the narrowing being progressively increasing between *Santanachelys*, *Rhinochelys*, *Desmatochelys*, and *Protostega* (see Hirayama, 1995). Also, the pterygoids are as narrow in the cheloniid *Allopleuron* as in the protostegid *Desmatochelys*. The difference is in the morphology of the narrowing, realized along a short distance on the pterygoid in *Allopleuron* whereas it extends on a long distance in protostegids.

**26. *Processus trochlearis oticus*. 0: with large contribution from quadrate; 1: with small contribution from quadrate.** KL state: ‘The states in H26 appear to have been reversed; this is corrected here’. This character has been modified here. It cannot be strictly applied to *Proganochelys*, in which a defined *trochlea otica* is lacking. Anyway, the muscles run on this area where the quadrate is wide, according to Gaffney (1979, figs. 16, 27, 42). We agree that the quadrate is primarily wider than the prootic in this area, but the prootic may be alternatively extended dorsally and not ventrally.

**27. *Processus trochlearis oticus*. 0: with prominent contribution from prootic**; **1: with small contribution from prootic (H27).** Modified from KL. This character coding seems in contradiction with the previous one. Alternatively it could be understood that, when the *trochlea* is clearly prominent, this prominence exhibits a strong or short participation of the prootic, independent to that of the quadrate, visible dorsally. The trochlea prominence is very variable according to taxa and may be more or less developed, or not at all, in width, or in length, or both in width and length; it could be also more prominent beside the cerebral cavity wall or beside the lateral external skull side, or both. Independently, the quadrate surface (between the cerebral cavity and the lateral margin of the skull) is reduced or not reduced dorsally and/or ventrally. This is an important point because it is supposed to be the only unambiguous synapomorphic character linking *Ocepechelon* and *Dermochelys*. This raises a problem, as the morphology of the trochlea is completely different in these taxa (with a trochlea prominence area not ossified in *Dermochelys*) (see above Results of the analysis).

***28. *Crista supraoccipitalis*. 0: small, not greatly extending beyond occipital condyle**; **1: large, greatly extending beyond occipital condyle (H28).** It would be also interesting to quantify the development of the *crista supraoccipitalis* in relation to that of the squamosal. The end of the *crista supraoccipitalis* is broken in *Ocepechelon* so that it has been coded 28/?, even if it was most probably short according to the surrounding bones morphology.

***29. *Foramina anteriora canalium carotici interni*. 0: widely separated; 1: close together (H29).**

***30. Internal carotid artery (posterior to junction with palatine artery). 0: not embedded in braincase elements**; **1: partially embedded**; **2: fully embedded (H30).** We have interpreted this character as the way the carotid enters the skull and is imbedded in bone as soon as it appears on the ventral face of the skull (meaning in Hirayama, 1998): 0: not embedded (= it enters the skull in the basisphenoid, but no distinction here if it enters in the basisphenoid medially or at the basisphenoid-pterygoid boundary); 1: partially embedded in an incomplete pterygoid canal (before entering in the basisphenoid close to the *sella turcica* with two foramina: a *foramen caroticum basisphenoidale* anteriorly and *a foramen posterius canalis caroti interni* posteriorly); 2: fully embedded by complete ventral closure of the canal (and *foramen posterius canalis caroti interni* located at the posterior extremity of the pterygoid). But the lower border of the canal can be so thin, for example in *Plesiochelys*, that it is ventrally either damaged, or incomplete, or complete, inducing both codings 0 and 1. The inner skull of *Dermochelys* remains so cartilaginous that the dorsal cover of the carotid is not ossified and not separated from the *canalis cavernosus* (looking not fully embedded in bone), whereas it is well covered ventrally, with the ‘canal’ beginning at the posterior border of the pterygoid (coded 2).

***31. Ventral surface of basisphenoid. 0: without V-shaped crest**; **1: with V-shaped crest (H31).**

**32. Basipterygoid processes of basisphenoid. 0: no basipterygoid projection; 1: basipterygoid posterolateral projections inducing a V shaped basisphenoid (H32).** Modified from KL, returning to a definition closer to Hirayama, 1998.

***33. *Dorsum sellae*. 0: low; 1: high (H33).** The definition of Hirayama is: ‘dorsum sellae high and separated from *sella turcica* and *foramen canalis carotici interni*’. KL statement is: ‘Hirayama also invoked separation from various braincase floor structures as part of this character; however these are all a product of the dorsum sella’s height’. In 1995, Hirayama’s definition adds that, when high, the *dorsum* *sellae* does not conceal the posterior portion of the *sella turcica*; effectively, when the *dorsum sellae* is high, there is a sagittal crest in a relatively long and elevated space separating the *sella* and the highest posterior border of the *dorsum sellae*, as in extant Cheloniidae. The *dorsum sellae* can be less high and the space shorter (*Euclastes*, *Erquelinnesia*, *Ocepechelon*) but an oblique to vertical crest is well present in all Chelonioidea we have observed and the *sella turcica* does not posteriorly extend below the *dorsum sellae*. The character has to be redefined with a new observation of specimens.

**34. *Rostrum basisphenoidale*. 0: wide, trabeculae separate; 1: trabeculae approximated or fused in a rod-like structure (H34).** KL state: ‘Hirayama’s (1998) original character (rod-like rostrum present/absent) lumped together taxa that had either a wide rostrum, or no rostrum at all (rod-like rostrum absent). Under the new phrasing here, taxa with reduced rostrums (see next character) are coded as inapplicable’. Definition of state 1 is modified as follows: the trabeculae are often close together without being fused in a rounded rod like structure, forming rather a double flattened stick.

**35. *Rostrum basisphenoidale*. 0: prominent**; **1: reduced (H35).** The primitive state of absence of prominence is not separated from the state 1 of reduction by lack of ossification, as in *Dermochelys.*

***36. Junction of palatine artery and internal carotid artery. 0: not enclosed in bone**; **1: enclosed in bone (H36).** Unknown in *Ocepechelon*.

***37. *Foramen caroticum laterale*. 0: not larger than the *foramen anterius canalis carotici interni*; 1: larger (H37).** Unknown in *Ocepechelon*.

***38. Foramen caroticum laterale. 0: not confluent with the *canalis cavernosus***; **1: confluent (H38).** Unknown in *Ocepechelon*.

**39. Mandible. 0: with narrow triturating surface, symphysis less than 1/3 of jaw length**; **1: with broad triturating surface, symphysis >1/3 jaw length.** From the skull anatomy of *Ocepechelon,* it is obvious that the symphysis was long and narrow and slightly higher than half the jaw length. It is an autapomorphy. In the absence of preservation of the mandibule, we have not coded the character for *Ocepechelon*.

***40. Dentary. 0: not hooked; 1: hooked (H40).** *Ocepechelon* had probably a spatulated lower jaw, without hook, but it may be hypothesized that the spatula is a derivation from a hook. We have coded it as inapplicable. European Oligocene Chelydridae lack a hooked dentary (coded 0).

***41. Symphyseal ridge of dentary. 0: absent; 1: present, but not exposed laterally; 2: present and greatly developed, being exposed laterally.** We agree with the combination of characters H41 and H42 by KL. Not preserved in *Ocepechelon*, nor the following characters.

***42. Lingual ridge of dentary. 0: prominent**; **1: weak or absent (H43).**

***43. Dentary expanded posteriorly, almost reaching articular surface. 0: no. 1: yes.** We agree with the modification of H44 by KL.

***44. Splenial. 0: present**; **1: absent (H45).**

***45. Transverse process of cervicals. 0: double; 1: single (H46).**

***46. Shape of central articulation of posterior cervicals. 0: as high as wide; 1: much wider than high (H47).**

***47. Ventral keel on posterior cervical centra. 0: absent; 1: present. (H48).**

***48. Cervical central articulations. 0: amphicoelous; 1: convex-concave (H49).**

H coded 49/? and KL coded 48/? for *Notochelone*. However, Gaffney (1981) mentioned the specimen QM F10619, from the type series attributed to *Notochelone*, which includes hyoplastron, scapula, and vertebral fragments with an amphicoelous cervical.

***49. Biconvex anterior cervical centrum 0: absent**; **1: present (H50 part).**

***50. Biconvex anterior cervical centrum. 0: on 2nd or 3rd cervical**; **1: on 4th cervical (H50 part).**

***51. 8th cervical. 0: amphicoelous; 1: procoelous; 2: biconvex; 3: opistocoelous (H51).**

***52. 8th cervical centrum. 0: not shorter than 7th**; **1: shorter than 7^th^ (H52).**

***53. Double cervical articulation between 7th and 8th cervicals. 0: absent. 1: present (H53).**

***54. 1st thoracic vertebra, anterior articulation. 0: facing anteriorly**; **1: facing ventrally or anteroventrally (H54).**

***55. 1st thoracic rib. 0: long, distal end extending to lateral margin of 1st costal**; **1: short, distal end does not extend beyond nuchal (H55).** In absence of postcranial elements for *Ocepechelon,* the definition is preserved. However**,** the analysis is incomplete and the state 1 should be replaced by several states; the first thoracic rib can be: half reduced in width, parallel to the second one and laterally free (extant Cheloniidae); originating from rib 2, not completely reduced in width and thickness (for example Chelydridae); issued from rib 2, not completely reduced in width but well reduced in thickness and distinctly arched (for example in Trionychidae); issued from rib 2, reduced in width and thickness (other Chelomacryptodira).

***56. 10th thoracic rib. 0: contacting 8th pleural; 1: ends freely (H56).** We have not observed state 0 in Chelydridae (individual or taxonomic variability?).

***57. Chevrons. 0: present; 1: absent (H57).**

***58. Anterior part of tail. 0: amphicoelous vertebrae**; **1: procoelous vertebrae (H58).** We return to a definition closer to that of Hirayama (“portion” for “part”, “caudals” for “tail”). Chelydridae are correctly coded 1 (see below).

**59. Posterior part of tail. 0: amphicoelous**; **1: opisthocoelous**; **2: procoelous vertebrae (H59).** Same as above, character less clearly defined by KL compared to Hirayama in which it was: posterior portion (part) of caudals (tail) (with): amphicolous, opisthocoelous or procoelous. Procoely demonstrates a progression in turtle evolution. Primitively the tail includes: 1) amphicoelous (both surfaces weakly concave) vertebrae (*Proganochelys*: Gaffney, 1990; Baenidae: Brinkman, 2003); 2) one platyconcave vertebra - others opisthocoelous, or only opisthocoelous vertebrae (*Meiolania*; Gaffney, 1985); 3) one platyconvex anterior vertebra - one biconcave (amphicoelous) vertebra - others opisthocoelous (*Chelydra*), or one platyconvex anterior vertebra – one or several (2 or 3) procoelous vertebrae – one biconcave (amphicoelous) vertebrae – others opisthocoelous (*Chelydra*, *Platysternon*; some extant and fossil pleurodirans including some Chelidae: Fuente et al., 2001, and *Dortoka*: Lapparent de Broin & Murelaga, 1999), plus sometimes toward the extremity of the tail: one biconvex vertebra - some procoelous ones (also *Chelydra*); 4) one platyconvex - procoelous vertebrae, or all procoelous vertebrae (all other extant turtles). This implies that Chelydridae must be coded (1, 2) according to the definition of Hirayama, once completed.

***60. Coracoid. 0: shorter than humerus**; **1: at least as long as humerus (H60).**

**61. Scapular angle, between scapular prong and acromion. 0: around 90 degrees; 1**: **from ca. 100 degrees and more.** We modified KL’s definition ‘scapular angle…at least 110 degrees’, itself modified from H61: ‘ scapular angle…about 110° and more’, because the angle is 100° in one specimen of *Lepidochelys* (MNHN), an extant cheloniids like *Chelonia* (the angle of which is just 110°); but it is 100°-110° in *Pupigerus* (IRScNB n°8632 and BM(NH) 28853); therefore both *Chelonia* and *Pupigerus* are coded 1. The limit between the two states is difficult to establish without an accurate specimen sampling. We do not know on the basis of which specimen *Euclastes* is coded, but the closely related *Erquelinnesia* has an angle about 95° (Zangerl, 1971); it is about 100° in *Toxochelys latiremis* (Everhart, 2010). Large angles distinctly above 110° (120º-130º) are found in chelonioids such as A*llopleuron*, *Archelon*, *Dermochelys*, and unpublished specimens from the Maastrichtian of Grand Daoui (see ‘Detailed description**’**).

***62. Lateral process of pubis (pectineal process). 0: small, not extending anteriorly beyond level of medial portion of pubis; 1: large, extending anteriorly beyond level of medial portion of pubis (H62).**

**63. Thyroid fenestra. 0: small to moderate, and subdivided by pubis-ischium contact**; **1: large and single (H63).** The polarity is reversed in KL analysis. The primitive pelvis has two small thyroid fenestrae well separated by bones (*Proganochelys*, *Palaeochersis* etc.). Dermochelyids (Gervais, 1872; Hirayama & Chitoku, 1996) and protostegids (Hay, 1908; Hirayama, 1998) have two small and well separated thyroid fenestrae. The thyroid fenestrae are small and more or less separated by bone in bottom walkers and terrestrial forms, but in fully aquatic swimming forms (Cheloniidae, Trionychia) (Zug, 1971) the fenestrae are large and shortly separated into a double fenestra or there is an unique wide fenestra. In living species, when the fenestra is large and not separated by bone, a cartilaginous septum remains, possibly a paedomorphic condition. *Euclastes* was coded by KL as possessing a single fenestra but it may be double because of the occurrence of a thin ossified septum in *Erquelinnesia* (Zangerl, 1971, pl. 9, fig. 2 - reference not cited by Hirayama). Similar large thyroid fenestrae united by a narrow ossified septum are present in *Allopleuron* (Mulder, 2003).

***64. Lateral process of ischium or metischial process. 0: prominent, extending laterally as wide as entire ischium; 1: small but distinct process; 2: rudimentary or lost (H64).** We agree, although the definition is not easy to understand.

***65. Large pelvis approaching coracoid. 0: no; 1: yes (H65).**

***66. Humerus. 0: shorter than femur**; **1: longer than femur (H66).** ***67. Lateral process of humerus. 0: abuts caput humeri; 1: slightly separated from caput humeri; 2: located distal to caput humeri but along proximal end of shaft; 3: located at middle of humeral shaft.**

***68. Proximal articular surface of humerus. 0: with shoulder on preaxial side, upturned; 1: without shoulder, not upturned (H68).**

***69. Scar for *M*. *latissimus dorsi* and *M*. *teres major*. 0: located anterior to humeral shaft**; **1: located at middle of shaft (H69).**

***70. Lateral process of humerus V-shaped. 0: no; 1: yes (H70).** (i.e. radial small trochanter). The character needs a better explanation.

***71. Expansion of lateral process of humerus limited to anterior surface of shaft. 0: no, expands onto ventral surface; 1: yes (H71**). Difficult to understand when comparing the taxa coded 1 to the others. The character should be better explained.

***72. Medial concavity of lateral process of humerus. 0: absent**; **1: present (H72).**

***73. Prominent anterior projection of lateral process of humerus. 0: absent**; **1: present (H73).**

**74. Ulna radius contact through rugosity and ridge. 0: absent**; **1: present (H74).** We agree for the marine forms, but this character is also present in terrestrial Testudinidae which are here considered as part of the Chelomacryptodira.

***75. Radius curves towards anterior. 0: no; 1: yes (H75).**

***76. Carpal and tarsal elements. 0: not flattened; 1: flattened (H76).**

***77. 3rd to 5th digits modified into paddle with rigid articulations. 0: no; 1: yes (H77).**

***78. 1st and 2nd digits modified into paddle with rigid articulations. 0: no; 1: yes (H78).** We agree, but this character is not so obvious for the rigidity of 1st digit in extant Cheloniidae.

***79. Femoral trochanters. 0: distinct, and separated from one another**; **1: indistinct and connected by bony ridge** (i.e. fused trochanters) **(H79).** There is a functionnal convergency between Chelonioidea and terrestrial Testudinidae among Chelomacryptodira (Renous et al., 2008).

***80. Scute sulci on carapace. 0: prominent; 1: absent or poorly developed (H80).** See remarks on characters 1 and 91.

***81. Posterior nuchal fontanelles. 0: absent; 1: present (H81).** Specific for *Ctenochelys* and *Toxochelys* in the H and KL matrix; however posterior nuchal fontanelles are also present in *Erquelinnesia* (Zangerl, 1971) (coded ‘?’ in *Euclastes*).

**82. Cervical scute. 0: overlying less than half width of nuchal; 1: overlying more than half width of nuchal (H82).** This character is incompletely defined. Primitive turtles (i.e. *Proganochelys*) have a wide nuchal and a wide cervical, the cervical slightly narrower than the half nuchal width. The trend is to the reduction of both nuchal and cervical. However, their respective narrowings are not linked (in various Chelomacryptodira, the cervical becomes highly reduced or disappears) so that the correlation nuchal-cervical in the definition of the character is not the best choice with the Chelomacryptodira included in the matrix. Beside, various ingroup taxa (*Puppigerus*), as well as some outgroup ones (Chelydridae, Plesiochelyidae) have the cervical width and half-nuchal width equal: another intermediate state should be proposed here. The Plesiochelyidae have the cervical fragmented into three well individualized scutes (and well distinct from that of the eurysternid *Palaeomedusa*, as figured in Joyce, 2003), and the entire cervical block width is wider, equal, or narrower than the half nuchal width; it is therefore coded 0 as in H and KL because it corresponds to the primitive state in the family.

***83. 1st vertebral. 0: not reaching 2nd marginal; 1: reaching 2nd marginal (H83).**

***84. Thick neurals with median keel. 0: no; 1: yes (H84).**

***85. Neural number. 0: eight or less; 1: nine; 2: ten or more (H85).** Not applicable to *Dermochelys*.

***86. Neural shape. 0: mostly hexagonal with short anterior or posterior sides; 1: mostly hexagonal, equally sided; 2: mostly rectangular (H86).** We have restaured the word ‘mostly’ of Hirayama & Chitoku (1996). It should be noted that, with the addition of pentagonal and octogonal neurals, some taxa such as Chelydridae (fossil and extant), *Platysternon*, and most Testudinidae (Chelomacryptodira) remain difficult to code. According to the most primitive condition in Oligocene *Chelydropsis,* Chelydridae are coded 0 like Chelomacryptodira. Not applicable to *Dermochelys*.

***87. Neural reduction. 0: all neurals present; 1: 7th and 8th neurals reduced or lost; 2: all neurals lost (H87).** We agree, but there are intermediate conditions between all neurals present and no neurals (*Dermochelys*). In many turtles (including Chelomacryptodira), neurals additional to 7 and 8, but not all, disappear as a result of dorsally meeting pleurals.

***88. Suprapygals. 0: 1st suprapygal moderately large; 1: 1st suprapygal absent or much smaller than 2nd (H88).**

***89. Pleural reduction. 0: all present; 1: more than half of pleurals retained; 2: pleurals greatly reduced; 3: pleurals absent (H89).** The character concerns the degree of reduction of the dermal ossification embedding the ribs in the pleurals, from vertebrae to marginally, i.e. the importance of the peripheral-pleural fontanelles, and not the reduction of the number of the pairs of pleurals.

**90. 9th pleural plate. 0: present; 1: absent (H90).** Modified from KL (reversed polarity): a ninth pleural is primitively present in *Proganochelys* and in Mesozoic taxa, sometimes retained in marine forms.

***91. Plastral scute sulci. 0: present; 1: absent or rudimentary (H91).** We agree, but see comments on characters 1 and 80.

**92. Axillary buttress. 0: only reaching peripheral; 1: reaching past peripheral, onto 1st pleural (H92).** Modified from KL (inversed polarity) and coded as in Hirayama & Chitoku (1996). Not applicable to *Dermochelys*.

**93. Inguinal buttress. 0: only reaching peripheral; 1: reaching past peripheral, onto 5th or 6th pleurals (H93)**. Modified from KL (inversed polarity). Not applicable to *Dermochelys*.

***94. Femoroanal anal sulcus. 0: not reaching hypoplaston. 1: reaching hypoplastron (H94).** Not applicable to *Dermochelys*.

***95. Plastral index. 0: larger than 100; 1: between 100 and 60. 2: lower than 60.**

**(H95).** The definition is found in Hirayama & Chitoku (1996), but not illustrated.

***96. Plastron. 0: not star-shaped; 1: star-shaped (H96).** From the author’s coding, we suppose that ‘star-shaped’ applies to equally long and wide indented hyo- and hypoplastron, resulting in a massive, roughly squared mid-plastron, such as that of advanced protostegids.

***97. Plastral fontanelles between hyo- and hypoplastra. 0: absent; 1: smaller than hyo- or hypoplastron; 2: at least as large as hyo- or hypoplastron (H97).** We agree, but this character does not give a lot of information concerning the different fontanelle shape patterns.

***98. Epiplastra. 0: wide; 1: narrow (H98).** We agree as far as the anterior part of the epiplastra is concerned. Indeed, they can be independently secondarily widened anteriorly in the late Tertiary European Chelydridae and in *Platysternon*.

***99. Entoplastron tightly sutured with hyoplastron. 0: yes; 1: no (H99).**

***100. Epi- and entoplastron. 0: separate; 1: fused (H100).**

***101. Entoplastron. 0: without elongate lateral wing; 1: with elongate lateral wing (H101).** We disagree with the coding by H and KL; state 1 was applied to Protostegidae and *Mesodermochelys*, but is, in our opinion, only to be applied in advanced Protostegidae. Not preserved in *Ocepechelon* and not applicable to *Dermochelys*.

***102. Xiphiplastra. 0: wide; 1: narrow (H102).** We agree, but it should be necessary to distinguish two possibilities. The primitive posterior lobe of the xiphiplastron is wide at the level of the inguinal notch and progressively narrowed posteriorly, with lateral borders converging and without anal notch. When the xiphiplastra are narrow, they might be present in: 1) derived taxa with a cruciform plastron (the whole xiphiplastron is present and narrowed without intermediate fontanelle, such as in Sinemydidae and Chelydridae); 2) in taxa with important medial fontanelles where only the lateral part of the xiphiplastron is ossified, for example in marine forms such as *Allopeuron*, *Protosphargis*, Protostegidae and *Dermochelys*. These two conditions are not homologous, which makes the character coding poorly informative. The following character (103) is more relevant, but may also be more or less confusing for the same reason.

***103. Medial contact of xiphiplastra. 0: sutured along its entire edge; 1: reduced or lost (H103).**

***104. Short xiphiplastra with lateral curvature. 0: absent; 1: present (H104).** We agree, but the limit between ‘with’ and ‘without’ is not evident in some taxa (i.e. *Santanachelys*).

**5. Character matrix**

0, primitive state; 1, 2, 3 derived states; numbers between brackets, polymorphism; ?, missing character; -, not applicable.

**Hypothetical outgroup**

0000000000 0000000000 0000000000 0000000000 0000000000 0000000000 0000000000 0000000000 0000000000 0000000000 0000

**Plesiochelyidae**

0011111101 1100000011 1000000001 00100(01)0000 100010000- 000000?000 00100?0000 0000000000 0000000001 0110000000 0000

***Xinjiangchelys***

????01?2?? ?????????? ????0001?1 00???0??00 00??10000- 1000000110 0010000000 00000?0000 00(01)0000001 0001100000 0000

**Sinemydidae**

1(01)(01)1111200 01000?0000 0000000101 ?00?100000 0101000111 (12)000010110 00100(01)0000 0000000001 (01)(01)10(01)(02)0(01) (01)0 (01)001(12)0(01)1(01)0 0100

**Chelydridae**

0110111200 0100000000 0000000102 0000010000 0101111111 11110(01)01(12)0 00(01)0000000 0000000000 0000000001 000(01)(12)01110 0100

**Chelomacryptodira**

(01)11(01)(01)11(02)0(01) (01)(01)00(01)(01)000(01) (01)00000010(12) 000001(01)0(01)(01) (012)(01)(01)111(01)11(01) (123)111(01)1112(01) 00(01)00(01)(01)0(01)0 00000000(01)(01) 00(01)(01)00(01)(01)00 (01)(01)(01)0(012)0(01)(01)00 0(01)(01)0

***Toxochelys***

?111100100 0101100100 0000000112 1010010000 (01)101111111 1101??1121 0010011000 0001011000 1100100011 ?00?201110 0100

***Ctenochelys***

?111100000 0101110100 0010000112 1111010000 1101111111 1001101121 0010011000 0001011000 11?1110011 ?00?201110 0100

***Euclastes***

0111100000 0001111100 2010000112 1111011010 0101?????? ?????????0 00000????? 00010???00 ???0?????? ?00?201110 0110

***Puppigerus***

0111000000 0001111100 2010000112 1111011010 1101111111 1111101121 1011012101 0001011110 0100100001 000?101110 0100

***Chelonia***

0111000000 0001111100 2010000112 1111011100 2001111111 1111101121 1011012111 0001011110 0110100011 0001001110 0110

***Bouliachelys***

?01000?001 1011100011 100000011? 10110?0000 ?????????? 1101?????? ?????????? ?????????? ?????????? ?????0???0 ????

***Notochelone***

0010001011 1001010011 1001000110 0011010000 110110???? ?????????1 1???012100 11001111?0 0?01020011 100?001110 ?111

***Santanachelys***

0001001011 1101100001 1001000110 0011010000 1101100110 10000?11?1 110?012100 10001110?0 0101020111 000?001110 1100

***Desmatochelys***

00010011?1 1001010011 1001110110 0011010000 1101100110 10001????1 1101012100 1100111110 0110020111 100?002110 1?1?

***Chelosphargis***

00000?10?1 1?0101001? ?001??0110 0011010000 11??1001?? ?????????1 1???012110 110??????0 0?01020111 100?011110 1111

***Protostega***

1110000011 101101?011 2101110110 0011010000 0101100110 1000101121 1101112110 1100111111 0??1120120 100?011111 1111

***Corsochelys***

1??0?01201 1????????? ?000001112 001-1110?? ????10000- ??00?????1 100?01210? 0000011?11 0010020011 100?002110 ?110

***Mesodermochelys***

?????????? ?????????? ???0?010?? ????????00 2011100111 1000101121 1101012100 0010?11111 0??0021111 100?002110 1110

***Dermochelys***

1110000001 1111111(01)00 2100001012 001-111101 0111110111 1000001121 1101013110 0010011111 ------2-3- 1---002110 -110

***Ocepechelon***

1001110001 1001000001 2100001?12 00010????- ?????????? ?????????? ?????????? ?????????? ?????????? ?????????? ????

Additional references

Anquetin J., Barrett P.M., Jones M.E.H., Moore-Fay S. & Evans S.E. (2009). A new stem turtle from the Middle Jurassic of Scotland: new insights into the evolution and palaeoecology of basal turtles. *Proc. R. Soc. London* B 276 (1658), 879–86.

Anquetin J. (2010). The anatomy of the basal turtle *Eileanchelys waldmani* from the Middle Jurassic of the Isle of Skye, Scotland. *Earth Env. Sc. Trans. R. Soc. Edinburgh* 101, 67–96.

Bardet N., Pereda Suberbiola X, Jouve S., Bourdon E., Vincent P., Houssaye A., Rage J.C., Jalil N.-E., Bouya B. & Amaghzaz M. (2010). Reptilian assemblages from the latest Cretaceous – Palaeogene phosphates of Morocco: from Arambourg to present time. *Hist. Biol.* 22 (1-3), 186-199.

Bardet N., Gheerbrant E., Noubhani A., Cappetta H., Jouve S., Bourdon E., Pereda Suberbiola X., Jalil N.-E., Vincent P., Houssaye A., Solé F., El Houssaini Darif Kh., Adnet S., Rage J.-C., Lapparent de Broin F. de, Sudre J., Bouya B., Amaghzaz M. & Meslouh S. Les Vertébrés des Phosphates crétacés-paléogènes (70,6 – 46,6 Ma) du Maroc. (in press) *Mém. Soc. Géol. France.*

Bour R. & Dubois A. (1986). Nomenclature ordinale et familiale des Tortues (Reptilia). Note complémentaire. *Bull. mens. Soc. Lin. Lyon* 55 (3), 87-90.

Brinkman D.B. (2003). Anatomy and systematics of *Plesiobaena antiqua* (Testudines; Baenidae) from the mid-Campanian Judith River Group of Alberta, Canada. *J. Vert. Pal*. 23 (1), 146–155.

Brinkman D.B., Aquillon-Martinez M. C., De Leon Da Vila C.A., Jamniczky H., Eberth D. A., & Colbert M. (2009). *Euclastes coahuilaensis* sp. nov., a basal cheloniid turtle from the late Campanian Cerro del Pueblo Formation of Coahuila State, Mexico. *PaleoBios* 28, 76–88.

Brinkman D.B. & Peng J.H. (1993a). *Ordosemys leios*, n.gen., n.sp., a new turtle from the early Cretaceous of the Ordos Basin, Inner Mongolia. *Can. J. Earth Sc*. 30 (10/11), 2128-2138.

Brinkman D.B. & Peng J.H. (1993b). New material of *Sinemys* (Testudines, Sinemydidae) from the Early Cretaceous of China. *Can.* *J. Earth Sc*. 30 (10/11), 2139-2152.

Broin F. de. (1977). Contribution à l'étude des Chéloniens. Chéloniens continentaux du Crétacé et du Tertiaire de France. *Mém. Mus. natn. Hist. nat.* Paris C 38, i-ix, 1-366.

Broin F. de & Fuente M.S. de la (1993). Les tortues fossiles d'Argentine. *Ann. Pal.* 79 (3), 169-231.

Capellini G. (1884). Il chelonio veronese (*Protosphargis veronensis* Cap.) scoperto nei 1852 nel cretaceo superiore presso S. Anna di Alfaedo in Valpolicella. *Mem. R. Acad. Lincei. Clas. Sci. Fis. Mat*. 18, 291-320.

Cigala Fulgosi F., Kotsakis T., Massari F., Medizza F. & Sorbini L. (1980). Il giacimento di S. Anna d’Alfaedo. In: “ I vertebrati fossili italiani” Catalogo della Mostra, Verona, 123-127.

Everhart M. (2010). Marine turtles from the Western Interior Sea. *w*ww.oceansofkansas.com/Turtles.html

Fernández M.S. & Fuente M.S. de la (1993). Las tortugas casiquelidias de las calizas litograficas titonianas del area Los Catutos, Neuquén, Argentina, *Ameghiniana* 30, 283-295.

Fuente M.S. de la & Fernández M.S. (2011). An unusual pattern of limb morphology in the Tithonian marine turtle *Neusticemys neuquina* from the Vaca Muerta Formation, Neuquén Basin, Argentina. *Lethaia*, 44 (1), 15-25.

Fuente M. de la, Lapparent de Broin F. de & Manera de Bianco T. (2001). The oldest and first nearly complete skeleton of a chelid, of the *Hydromedusa* sub-group (Chelidae, Pleurodira), from the Upper Cretaceous of Patagonia. *Bull. Soc. Géol. France* 172 (2), 105-112.

Gaffney E.S. (1975a). A Taxonomic Revision of the Jurassic Turtles *Portlandemys* and *Plesiochelys*. *Amer. Mus. Nov*. 2574, 1-19.

Gaffney E.S. (1975b). *Solnhofia parsonsi*, a new Cryptodiran Turtle from the Late Jurassic of Europe. *Amer. Mus. Nov*. 2576, 1-25.

Gaffney E.S. (1975c). Phylogeny of the Chelydrid Turtles: A Study of shared derived Characters in the Skull. *Fieldiana, Geol*. 33 (9), 157-178.

Gaffney E.S. (1976). Cranial Morphology of the European Jurassic Turtles *Portlandemys* and *Plesiochelys*. *Bull. Amer. Mus. Nat. Hist*. 157 (6), 487-544.

Gaffney E.S. (l979). Comparative Cranial Morphology of Recent and Fossil Turtles. *Bull. Amer. Mus. Nat. Hist.* l64 (2), 65-376.

Gaffney E.S. (1981). A Review of the Fossil Turtles of Australia. *Amer. Mus. Nov*. 2720, 1-38.

Gaffney E.S. (1983). The cranial morphology of the extinct horned turtle, *Meiolania platyceps*, from the Pleistocene of Lord Howe Island, Australia. *Bull. Amer. Mus. Nat. Hist.* 175 (4), 361-480.

Gaffney, E.S. (1984). Historical analysis of theories of chelonian relationships. *Syst. Zool*. 33, 283-301.

Gaffney E.S. (1985). The Cervical and Caudal Vertebrae of the Cryptodiran Turtle, *Meiolania platyceps*, from the Pleistocene of Lord Howe Island, Australia, *Amer. Mus. Nov*. 2805, 1-29.

Gaffney E.S. (1990). The comparative osteology of the Triassic turtle *Proganochelys*. *Bull. Amer. Mus. Nat. Hist.* 194, 1-263.

Gaffney E.S. (1996). The postcranial morphology of *Meiolania platyceps* and a review of the Meiolaniidae. *Bull. Amer. Mus. Nat. Hist.* 229, 1-165.

Gaffney E.S., Hutchison J.H., Jenkyns F.A. & Meeker L.J. (1987). Modern Turtle Origins: The Oldest Known Cryptodire. *Science* 237, 289-291.

Gaffney E.S. & Jenkyns F.A. Jr. (2010). The cranial morphology of *Kayentachelys*, an Early Jurassic cryptodire, and the early history of turtles. *Acta Zool.* 91, 335-368.

Gaffney E.S., Kool L., Brinkman D.B., Rich T.H. & Vickers-Rich P. (1998). *Otwayemys*, a New Cryptodiran Turtle from the Early Cretaceous of Australia. *Amer. Mus. Nov*. 3233, 1-28.

Gaffney E.S. & Meylan P.A. (1988). A phylogeny of turtles. In: *The Phylogeny and Classification of the Tetrapods*. Vol. 1: Amphibians, Reptiles, Birds, M. J. Benton Ed., Systematics Association Special Volume 35A, Clarendon Press, Oxford 5, 157-219.

Gaffney E.S., Tong H., Meylan P.A. (2006). Evolution of the side-necked turtles: the families Bothremydidae, Euraxemydidae, and Araripemydidae. *Bull. Amer. Mus. Nat. Hist.* 300, 1-700.

Gaffney E.S. & Yeh X. (1992). *Dracochelys*, a New Cryptodiran Turtle from the Early Cretaceous of China. *Amer. Mus. Nov*. 3048, 1-13.

Gaillard C., Bernier P., Barale G., Bourseau J.-P., Lapparent de Broin F. de , Buffetaut E., Ezquerra R., Gall J.-C, Renous S. & Wenz S. (2003). A giant Upper Jurassic turtle revealed by its trackways. *Lethaia* 36, 315-322.

Gervais P. (1872). Ostéologie de SPHARGIS LUTH. *Nouv. Arch. Muséum, Mém.* 8, 199-228.

Gmira S. (1995). Etude des Chéloniens fossiles du Maroc. Anatomie, Systématique, Phylogénie. *Cahiers de paléontologie,* CNRS, Paris.

Gray J.E. (1869). Notes on the families and genera of Tortoises and on characters afforded by the Study of their Skulls. *Proc. Zool. Soc. London* 12, 165-225.

Hay O.P. (1908). The fossil turtles of North America. *Carnegie Inst. Washington Publ.* 75, 1-568.

Hirayama R. (1985). Cladistic analysis of Batagurine turtles (Batagurinae: Emydidae: Testudinoidea). A preliminary result. *Stud. Geol. Salm*., Vol. sp. 1. *Stud. Paleochel*. 1, 141-157.

Hirayama R. (1992). Humeral morphology of chelonioid sea-turtles; its functional analysis and phylogenetic implications. *Bul. Hobetsu Mus.* 8, 17–57 (in Japanese with English abstract).

Hirayama R. (1995). Phylogenetic systematics of chelonioid sea turtles. *The Island Arc* 3, 270-284.

Hirayama R. (1997). Distribution and diversity of Cretaceous chelonioids. In: *Ancient marine reptiles*, J. M. Callaway & E. L. Nicholls Eds., Academic Press, San Diego, 8, 225-241.

Hirayama R. (1998). Oldest known sea turtle. *Nature* 392 (6677), 705-708.

Hirayama R. & Chitoku T. (1996). Family Dermochelyidae (Superfamily Chelonioidea) from the Upper Cretaceous of North Japan. *Trans. Proc. Palaeont. Soc. Japan* N.S. 184, 597-622.

Hirayama R. & Tong H. (2003). *Osteopygis* (Testudines, Cheloniidae) from the Lower Tertiary of the Ouled Abdoun phosphate basin, Morocco. *Palaeontology* 46, 85-98.

Hooks III G.E. (1998). Systematic revision of the Protostegidae, with a redescription of *Calcaricheys gemma* Zangerl, 1953. *J. Vert. Pal.* 18, 85–98.

Jalil N-E., Lapparent de Broin F. de, Bardet N., Vacant R., Bouya, B., Amaghzaz M. & Meslouh S. (2009). *Euclastes acutirostris*, a new species of littoral turtle (Cryptodira, Cheloniidae) from the Palaeocene phosphates of Morocco (Oulad Abdoun Basin, Danian-Thanetian). *C.R. Palevol*. 8, 447-459.

Joyce W.G. (2003). A new Late Jurassic turtle specimen and the taxonomy of *Palaeomedusa testa* and *Eurysternum wagleri*. *PaleoBios* 23 (3), 1-8.

Joyce W.G. (2007). Phylogenetic relationships of Mesozoic turtles. *Bull. Peabody Mus. Nat. Hist.* 48, 3-102.

Joyce W.G. & Sterli J. (2010). Congruence, non-homology, and the phylogeny of basal turtles *Acta Zoo.* doi: 10.1111/j.1463-6395.2010.00491.x

Kear P. & Lee M.S.Y. (2006). A primitive protostegid from Australia and early sea turtle evolution. *Biol. Lett*. 2, 116-119.

Lapparent de Broin F. de (2000a). African chelonians from the Jurassic to the Present. A preliminary catalog of the African fossil chelonians. *Palaeont. Afr.* 36, 43-82.

Lapparent de Broin F. de (2000b). Les Chéloniens de Sansan. *Mém. Mus. natn. Hist. nat., Paris* 183 (11), 219-261.

Lapparent de Broin F. de (2001).The European turtle fauna from the Triassic to the Present. *Dumerilia* 4 (3), 155-216.

Lapparent de Broin F. de (2004). A new Shachemydinae (Chelonii, Cryptodira) from the Lower Cretaceous of Laos: preliminary data. *C. R. Palevol* 3, 387-396.

Lapparent de Broin F. de, Fuente M.S. de la & Fernández M. (2007). *Notoemys* (Chelonii, Pleurodira), Late Jurassic of Argentina: new examination of the anatomical structure and comparisons. *Revue Paléobiol.* 26 (2), 99-136.

Lapparent de Broin F. de, Lange-Badré B. & Dutrieux M. (1996). Nouvelles découvertes de tortues dans le Jurassique supérieur du Lot (France) et examen du taxon Plesiochelyidae. *Revue Paleobiol.* 15 (2), 533-570.

Lapparent de Broin F. de & Murelaga X. (1999). Turtles from the Upper Cretaceous of Laño (Iberian Peninsula). *Estud. Mus. Cienc. Nat. Alava* 14 (Num. Esp. 1), 135-211.

Lapparent de Broin F. de & Werner C. (1998). New late Cretaceous turtles from the Western Desert, Egypt. *Ann. Pal.* 84 (2), 131-214.

Lemell P., Beisser C.J., Gumpenberger M., Snelderwaard P., Gemel R. & Weisgram J. (2010). The feeding apparatus of *Chelus fimbriatus* (Pleurodira: Chelidae) – adaptation perfected ? *Amphibia-Reptilia* 31, 97-107.

Longman H.A. (1915). On a giant turtle from the Queensland Early Cretaceous. *Mem. Qld Mus.* 3, 24-29.

Lynch S.C. & Parham J. F. (2003). The first report of hardshelled turtles (Cheloniidae sensu lato) from the Miocene of California, including a new species (*Euclastes hutchisoni*) with unusually plesiomorphic characters. *Paleobios* 23, 21–35.

Mateus O., Jacobs L., Polcyn M., Schulp A., Vineyyard D., Buta Neto A. & Telles Antunes M. (2009). The oldest African eucryptodiran turtle: *Angolachelys ombeu*, from the Cretaceous of Angola. *Acta Pal. Pol.* 54 (4), 581–588.

Meylan P.A. & Gaffney E.S. (1992). *Sinaspideretes* is not the oldest trionychid turtle. *J. Vert. Pal.* 12, 257-259.

Młynarskii M. (1980). Die Schildkröten des Steinheimer Beckens. B. Chelydridae mit einem Nachtrag zu den Testudinoidea. *Palaeontographica* Suppl. 8, 2 (3), 1-35.

Mulder E.W.A. (2003). On the latest Cretaceous tetrapods from the Maastrichtian type area. *Natuurhist. Genootsch. Limburg* 44(1), 1-188.

Near T.J., Meylan P.A. & Shaffer H.B. (2005). Assessing Concordance of Fossil Calibration Points in Molecular Clock Studies: An Example Using Turtles. *Am. Nat*. 165 (2), 137-146.

Parham J.F. (2005). A reassessment of the referral of sea turtle skulls to the genus *Osteopygis* (Late Cretaceous, New Jersey, USA). *J. Vert. Pal*. 25, 71–77.

Parham J.F. & Fastovsky D.E. (1997). The phylogeny of Cheloniid Sea Turtles Revisited. *Chel. Conserv. Biol*. 2 (4), 548-554.

Parham J.F. & Hutchison J.H. (2003). A new eucryptodiran turtle from the Late Cretaceous of North America (Dinosaur Provincial Park, Alberta, Canada). *J. Vert. Pal.* 23, 783–798.

Parham J.F. & Pyenson N.D. (2010). New Sea Turtle from the Miocene of Peru and the Iterative Evolution of Feeding Ecomorphologies since the Cretaceous. *Jour. Pal*. 84 (2), 231–247.

Peng J. & Brinkman D.B. (1993). New material of *Xinjiangchelys* (Reptilia: Testudines) from the Late Jurassic Qiju Formation (Shishugou Group) of the Pingfengshan locality, Junggar Basin, Xinjiang. *Can. J. Earth Sc*. 30 (10/11), 2013-2026.

Renous S., Lapparent de Broin F. de, Depecker M., Davenport J. & Bels V. (2008). In *Biology of Turtles* (eds Wyneken, J., Godfrey, M.H. and V. Bels) CRC Press, Taylor & Francis Group, Boca Raton (FL) USA 5, 97-138.

Schumacher G.-H. (1973). In *Biology of Reptilia* Vol. 4 (eds Gans, C. & Parsons T. S.). Academic Press, New York.

Sterli J. & Joyce W. (2007). The cranial anatomy of the Early Jurassic turtle *Kayentachelys aprix Acta Pal. Pol*. 52 (4), 675-694.

Sukhanov V.B. (2006). An archaic turtle, *Heckerochelys romani* gen. et sp. nov., from the Middle Jurassic of Moscow region, Russia. *Fossil Turtle Res*. 1, 112-118.

Sukhanov V.B. (2000). Mesozoic turtles of Middle and central Asia. In. M.J. Benton, M.A. Shishkin, D.M. Unwin & E.N. Urochkin (eds). *The Age of Dinosaurs in Russia and Mongolia*, Cambridge University Press, 17, 309-367.

Sukhanov V.B. & Narmandakh P. (2006). New Taxa of Mesozoic Turtles from Mongolia. *Fossil Turtle Res.* 1, 119-127.

Tong H. & Hirayama R. (2004). First Cretaceous dermochelyid turtle from Africa, *Revue Paléobiol*. Vol spec. 9, 55-50.

Tong H. & Meylan P. (2013). Morphology and Relationships of *Brachyopemys tingitana* gen. et sp. nov. from the Early Paleocene of Morocco and Recognition of the New Eucryptodiran Turtle Family: Sandownidae. In: D.B. Brinman et al. (eds). *Morphology and Evolution of Turtles*, Vertebrate Paleobiology and Paleoanthropology, DOI:10.1007/978-94-007-4309-0_13, Springer Science+Business Media Dordrecht: 187-212.

Van Damme J. & Aerts P. (1997). Kinematics and Functional Morphology of Aquatic Feeding in Australian Snake-Necked Turtles (Pleurodira; Chelodina). *J. Morphol*. 233, 113-125.

Wieland G.R. (1909). Revision of the Protostegidae.  *Am. J. Sc.* Series 4, 27(158, 7), 101-130.

Wood R.C., Johnson-Gove J., Gaffney E.S. & Maley K.F. (1996).  Evolution and Phylogeny of Leatherback Turtles (Dermochelyidae), with Descriptions of New Fossil Taxa. *Chel. Cons. Biol*. 2 (2), 266-286.

Young C.C. & Chow M-C. (1953). New Fossil Reptiles from Szechuan China. *Acta Pal. Sin*. 1, 87-110.

Zangerl R. (1953a). The Vertebrate Fauna of the Selma Formation of Alabama. Part III. The turtles of the family Protostegidae. *Fieldiana, Geol. Mem*. 3, 59-133.

Zangerl R. (1953b). The Vertebrate Fauna of the Selma Formation of Alabama. Part IV. The turtles of the family Toxochelyidae. *Fieldiana, Geol. Mem*. 3, 137-277.

Zangerl R. (1960). The Vertebrate Fauna of the Selma Formation of Alabama. Part V. An advanced cheloniid sea turtle. *Fieldiana, Geol. Mem*. 3, 281-312.

Zangerl R. (1971). Two toxochelyid sea turtles from the Landenian sands of Erquelinnes (Hainaut) of Belgium. *Mém. Inst. R. Sc. Nat. Belg.* 169, 1-32.

Zangerl R. & Sloan R.E. (1960). A New Specimen of *Desmatochelys lowi* Williston, a primitive cheloniid sea turtle from the Cretaceous of South Dakota. *Fieldiana, Geol*. 14, 7-40.

Zug G.R. (1971). Buoyancy, locomotion, morphology of the pelvic girdle and hind limb and systematics of cryptodiran turtles. *Misc. Publ. Mus. Zool. Univ. Michigan* 142, 1-98.

Figure S1

Chosen examples of cladograms obtained from the modified Kear & Lee (2006) matrix [15].

S1.1: removing outgroups one after the other, except the hypothetical taxon (cladograms not presented here display only minor changes compared to the ones illustrated).


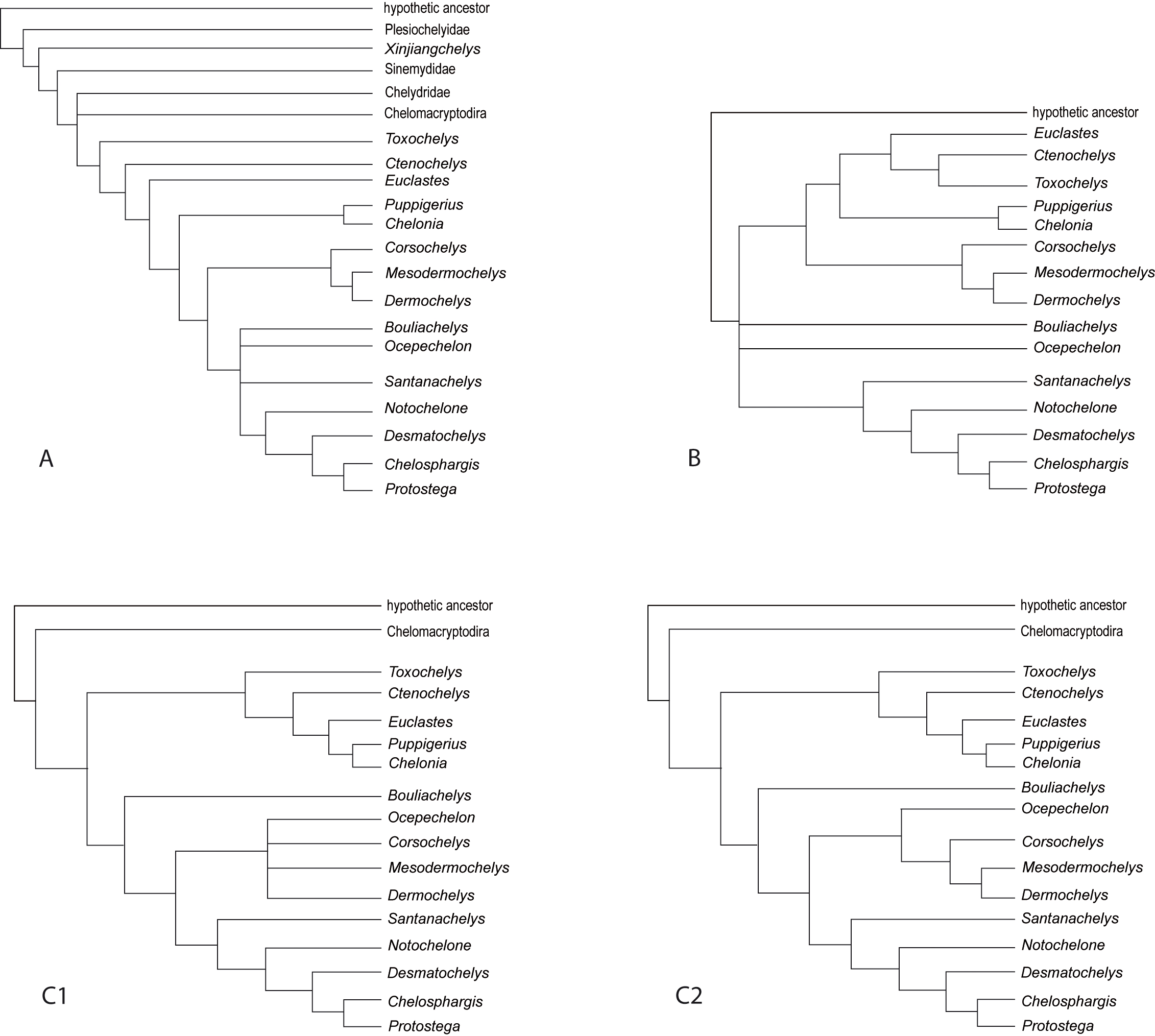


S1.2: Additional tree tested from the matrix of Figure S1.1 (C1 and C2), with the hypothetic taxon and Chelomacryptodira as outgroups. As in the strict consensus (Figure S1.1 - C1), L = 207, Ci = 54, Ri = 62.
